# MiR-138 is a potent regulator of the heterogenous *MYC* transcript population in cancers

**DOI:** 10.1038/s41388-021-02084-x

**Published:** 2021-12-22

**Authors:** Ng Desi, Velda Teh, Qing Yun Tong, Chun You Lim, Hossein Tabatabaeian, Xiao Hong Chew, Avencia Sanchez-Mejias, Jia Jia Chan, Bin Zhang, Priyankaa Pitcheshwar, Bei-En Siew, Shi Wang, Kuok-Chung Lee, Choon-Seng Chong, Wai-Kit Cheong, Bettina Lieske, Ian Jse-Wei Tan, Ker-Kan Tan, Yvonne Tay

**Affiliations:** 1 Cancer Science Institute of Singapore, National University of Singapore, Singapore, 117599 Singapore; 2 Department of Biochemistry, Yong Loo Lin School of Medicine, National University of Singapore, Singapore, 117597 Singapore; 3 Department of Surgery, Yong Loo Lin School of Medicine, National University of Singapore, Singapore, Singapore; 4 Department of Pathology, National University Health System, Singapore, Singapore; 5 Division of Colorectal Surgery, University Surgical Cluster, National University Health System, Singapore, Singapore; 6 Present Address: Department of Experimental and Health Sciences, Pompeu Fabra University, 08003 Barcelona, Spain

**Keywords:** Colorectal cancer, Oncogenes

## Abstract

3′UTR shortening in cancer has been shown to activate oncogenes, partly through the loss of microRNA-mediated repression. This suggests that many reported microRNA-oncogene target interactions may not be present in cancer cells. One of the most well-studied oncogenes is the transcription factor MYC, which is overexpressed in more than half of all cancers. *MYC* overexpression is not always accompanied by underlying genetic aberrations. In this study, we demonstrate that the *MYC* 3′UTR is shortened in colorectal cancer (CRC). Using unbiased computational and experimental approaches, we identify and validate microRNAs that target the *MYC* coding region. In particular, we show that miR-138 inhibits MYC expression and suppresses tumor growth of CRC and hepatocellular carcinoma (HCC) cell lines. Critically, the intravenous administration of miR-138 significantly impedes MYC-driven tumor growth in vivo. Taken together, our results highlight the previously uncharacterized shortening of the *MYC* 3′UTR in cancer, and identify miR-138 as a potent regulator of the heterogenous *MYC* transcript population.

## Introduction

More than half of protein-coding genes produce messenger RNA (mRNA) transcripts with alternative 3′ untranslated regions (UTRs), which contain binding sites for post-transcriptional regulators such as microRNAs (miRNAs). Alternative 3′UTRs thus comprise varying landscapes of miRNA response elements (MREs), enabling them to differentially regulate the stability and translational efficiency of their cognate mRNAs. In cancer cells, widespread shortening of 3′UTRs by alternative polyadenylation (APA) has been shown to contribute to tumorigenesis by controlling the expression of oncogenes in the absence of genetic alterations, in part by enabling them to escape miRNA-mediated regulation [1–4]. Critically, the vast majority of miRNA studies have focused on MREs located in the 3′UTRs of target mRNAs. The observation that 3′UTR shortening is increased in cancers suggests that many known miRNA:target interactions may be lost in cancer cells [3, 4], and highlights the need to better understand how established cancer driver genes with shortened 3′UTRs may be regulated in cancer cells.

In this study, we focus on the transcription factor MYC, which is overexpressed in more than half of all cancers [5, 6]. Intriguingly, although MYC is overexpressed 5 to 400-fold in 70% of colorectal carcinoma (CRC) patients and 4 to 6-fold in 44% of hepatocellular carcinoma (HCC) patients, genetic amplification of MYC is only detected in 10% and 4% of CRC and HCC patients respectively [7–11]. These observations that MYC overexpression is not always accompanied by underlying genetic aberrations suggest that post-transcriptional mechanisms may play key roles in regulating MYC expression and function in these cancers.

Previously, a *MYC* construct containing its 3′UTR was shown to have lower MYC protein expression compared to those without, demonstrating the importance of the 3′UTR in regulating MYC expression [12]. Multiple studies have identified miRNAs that bind to the *MYC* 3′UTR and regulate MYC expression [13–15]. In contrast, only a few miRNAs that target the *MYC* coding sequence (CDS) have been described. Specifically, miR-184 and miR-320b, as well as miR-320a and miR-744, were shown to inhibit MYC expression in CRC [16, 17] and HCC respectively [18, 19]. However, their potential efficacy as treatment options in MYC-driven cancers remains to be explored.

In this study, we find that the *MYC* 3′UTR is shortened in cancer, and integrate two complementary computational and biochemical approaches to identify miRNAs that target the *MYC* coding region as this is present in all *MYC* transcript variants. In particular, we show that miR-138 binds to the *MYC* CDS, inhibits MYC expression and suppresses tumor growth. Critically, the intravenous administration of miR-138 significantly impedes the tumor growth of MYC-driven mouse model. These results highlight the previously uncharacterized shortening of the *MYC* 3′UTR in cancer, and identify miR-138 as a potent regulator of the heterogenous *MYC* transcript population. These data may open new avenues for the treatment of MYC-driven cancers.

## Results

### Identification of potential MYC CDS-targeting miRNAs

**Fig. 1:**
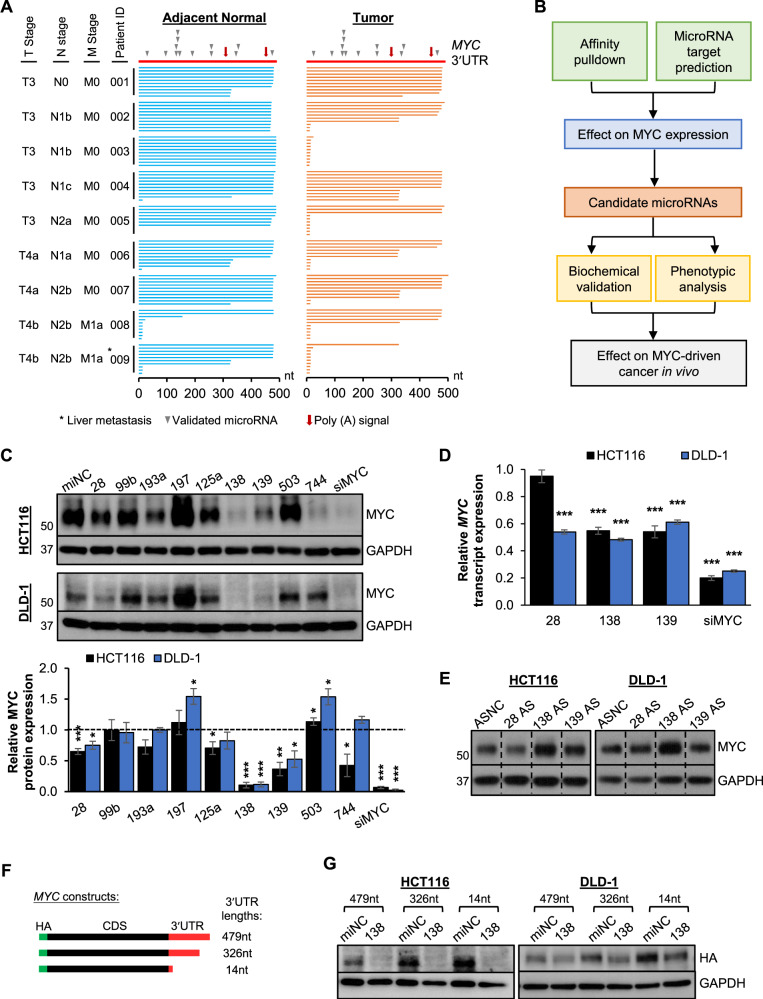
Identification of potential *MYC* CDS-targeting miRNAs. **A** Schematic representation of *MYC* 3′UTR isoforms detected by 3′ RACE in nine pairs of CRC tumor and adjacent normal patient samples (*N* = 9). **B** Flowchart outlining the workflow to identify and functionally characterize miRNAs that target the *MYC* CDS. MiRNAs were identified via affinity pulldown by MS2-tagged RNA Affinity Purification (MS2-TRAP) and miRNA target prediction by RNA22. **C** Western blot (top panel) and densitometry quantification (bottom panel) of MYC protein expression upon the overexpression of candidate miRNAs in HCT116 and DLD-1 cell lines. **D** RT-qPCR analysis of *MYC* transcript expression upon the overexpression of miR-28-5p, miR-138 and miR-139-3p in HCT116 and DLD-1 cell lines. **E** Western blot analysis of MYC protein expression upon miRNA inhibition by antisense miRNA inhibitors (AS) in HCT116 (left panel) and DLD-1 (right panel). **F** Schematic illustrating the HA-tagged MYC constructs with different 3′UTR lengths. **G** Western blot analysis of HA-tagged MYC protein expression upon miR-138 overexpression (138) in HCT116 (left panel) and DLD-1 (right panel). Mean ± SEM; *N* ≥ 3. *P < 0.05; **P < 0.01; ***P < 0.001.

The overwhelming majority of identified miRNA:MYC interactions are located in its 3′UTR. However, reports describing widespread 3′UTR shortening in cancers suggest that these interactions may not exist in cancer cells, contributing to the overexpression of MYC in multiple cancers often in the absence of genetic aberrations. To investigate this, we first examined potential APA of the *MYC* 3′UTR in tumor and adjacent normal samples from CRC patients (primary tumor stage (T stage) > 2) using 3′ Rapid Amplification of cDNA Ends (3′ RACE) (*N* = 9). Three main *MYC* 3′UTR isoforms were detected, two corresponding to annotated poly(A) signals and one in close proximity to the stop codon (Fig. 1A). Interestingly, more *MYC* 3′UTR shortening was observed in the tumor samples than in the adjacent normal samples, and in patients with cancer at the regional lymph nodes (N stage > 0). We postulate that *MYC* 3′UTR shortening will result in a loss of miRNA binding. In addition, this data suggests that studying the post-transcriptional regulation of the *MYC* CDS may lead to the identification of crucial miRNAs that modulate the expression of all *MYC* transcript variants, including those which are predominantly expressed in advanced cancer.

Two approaches were employed to systematically identify miRNAs that target the *MYC* CDS: affinity pulldown by MS2-tagged RNA affinity purification (MS2-TRAP) coupled with high-throughput qPCR and the miRNA target prediction by RNA22 (Fig. 1B) [20]. MiR-193a-5p, miR-197-3p, miR-28-5p and miR-99b-3p were shortlisted from the high-throughput qPCR analysis based on their consistent enrichment (>1.5) (Supplementary Fig. S1a and Supplementary Data File S1). The second shortlisting criterion was the downregulation of their expression in CRC (Supplementary Fig. S1b) as the MS2-TRAP was performed using the CRC cell line HCT116. Although the MS2-TRAP-qPCR approach allows for the identification of miRNAs which associate with the *MYC* CDS in a specific cell-type, it may not enrich for miRNAs that are lowly expressed in the particular cell line studied. As a complementary approach, we performed miRNA target prediction using RNA22. MiR-125a-3p, miR-138-5p (commonly known as miR-138), miR-139-3p, miR-503-5p and miR-744-5p (commonly known as miR-744) were shortlisted based on their predicted binding to conserved sites on both the human and mouse *MYC* CDS (Supplementary Table S1), as well as their downregulation in CRC (Supplementary Fig. S1c) and HCC (Supplementary Fig. S1d). As mentioned, miR-744 has been validated to inhibit MYC expression in HCC [19], therefore, it was included in this study as a positive control and to explore its regulation of *MYC* in CRC.

To study the effect of the shortlisted miRNAs on MYC expression, we overexpressed the individual miRNAs in HCT116 and DLD-1 which are commonly used CRC cell lines. In addition, both HCT116 and DLD-1 have elevated expression of MYC relative to two normal colon cell lines, CCD-18Co and CCD841CoN (Supplementary Fig. S1e). We found that only the overexpression of miR-28-5p (28), miR-138 (138) and miR-139-3p (139) consistently and significantly decreased MYC protein and transcript expression in both HCT116 and DLD-1 (Fig. 1C and D). Interestingly, these effects were not observed for miR-744, suggesting that its regulatory effect on MYC is cell-type specific. As a reciprocal approach, we next determined the effect of miR-28-5p, miR-138 and miR-139-3p inhibition on MYC expression using antisense miRNA inhibitors (AS). The inhibition of miR-138 significantly increased MYC protein expression, whereas miR-28-5p and miR-139-3p inhibition had no effect on MYC (Fig. 1E and Supplementary Fig. S1f). Based on these observations, we focused on miR-138 for subsequent experimental validation.

To investigate whether miR-138 could regulate *MYC* transcripts with varying 3′UTR lengths, we cloned the *MYC* 3′UTR isoforms downstream of the *MYC* CDS with a HA tag at the N-terminus (Fig. 1F). We found that miR-138 overexpression downregulated the expression of all HA-tagged *MYC* isoforms (Fig. 1G), demonstrating its ability to inhibit the expression of heterogenous *MYC* transcript isoforms.

### Validation of miR-138 as a MYC CDS-targeting miRNA

**Fig. 2:**
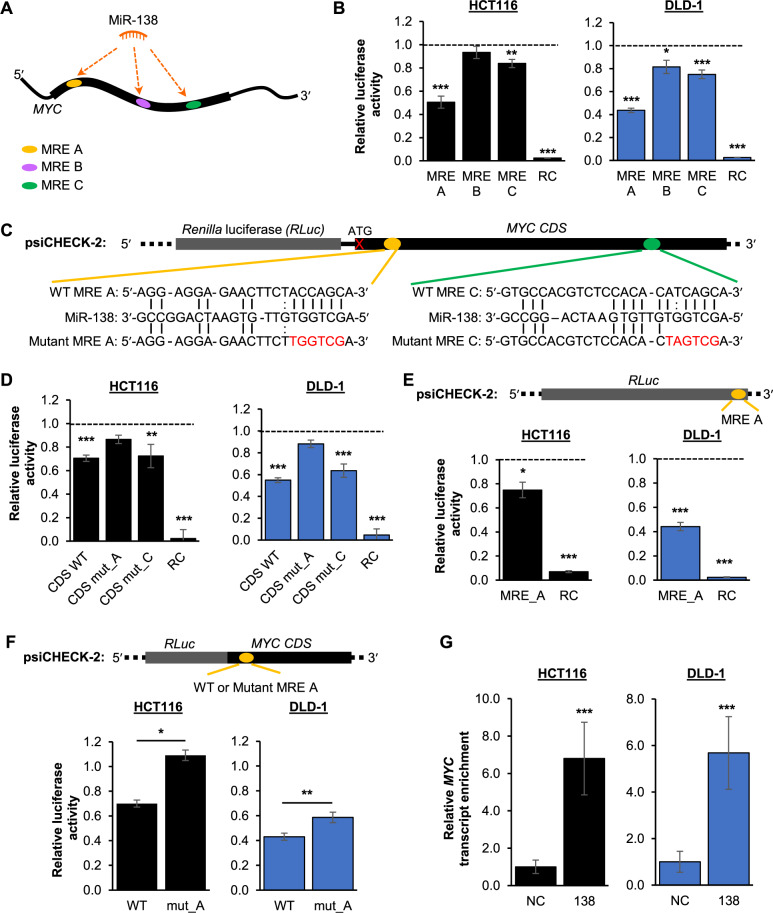
Validation of miR-138 as a *MYC* CDS-targeting miRNA. **A** Schematic representation of predicted miR-138 MREs on the *MYC* transcript. The thick line indicates the *MYC* CDS, while the thin lines indicate the UTRs. **B** Effect of miR-138 overexpression on the luciferase activity of the respective MRE-reporter constructs in HCT116 (left panel) and DLD-1 (right panel) cell lines. RC denotes the reverse complement of miR-138 which was used as positive control. **C** Schematic representation of the ATG-less *MYC* CDS cloned into the psiCHECK-2 vector. Location and sequences of the wild-type (WT) and mutant MREs A (yellow oval) and C (green oval) are depicted. Red font indicates the mutated nucleotides. The cross indicates the absence of the start codon ATG. **D** Effect of miR-138 overexpression on the luciferase activity of the *MYC* CDS reporter constructs with mutated MREs (CDS mut) compared to the wild-type CDS reporter (CDS WT) in HCT116 (left panel) and DLD-1 (right panel) cell lines. **E** Effect of miR-138 overexpression on the luciferase activity of the *Renilla* luciferase (*RLuc*) gene fused with MRE A in HCT116 (left panel) and DLD-1 (right panel) cell lines. Top panel illustrates the modification of *RLuc* gene construct. **F** Effect of miR-138 overexpression on the luciferase activity of the *RLuc* and *MYC* CDS fusion reporter construct with mutated MRE A (mut_A) compared to the wild-type *MYC* CDS (WT) in the HCT116 (left panel) and DLD-1 (right panel) cell lines. The top panel illustrates the fusion gene construct. **G** RT-qPCR analysis of *MYC* transcript enrichment upon biotinylated miR-138 pulldown in HCT116 (left panel) and DLD-1 (right panel) cell lines. (**B**, **D**, **E** and **F**) Mean ± SEM; *N* ≥ 3. **G** Mean ± STD; *N* ≥ 3. *P < 0.05; **P < 0.01; ***P < 0.001.

MiR-138 is predicted to have three MREs on the *MYC* CDS, denoted as MRE A, MRE B and MRE C (Fig. 2A and Supplementary Table S2). Both human and mouse miR-138 MREs are predicted to be at the same positions with similar nucleotide sequences, suggesting that miR-138-mediated regulation of the *MYC* CDS may be conserved in these two species (Supplementary Table S2). To validate these predicted MREs, we first performed MRE-luciferase reporter assays. The overexpression of miR-138 significantly and consistently decreased the relative luciferase activities of MRE A and MRE C, but did not have a consistent effect on MRE B in both HCT116 and DLD-1 (Fig. 2B). To further validate the regulation of the *MYC* CDS, we cloned the ATG-less *MYC* CDS downstream of the *Renilla* luciferase (*RLuc*) gene, after the stop codon. We also mutated the sequence corresponding to the miR-138 seed region within each MRE on a *MYC* CDS-luciferase reporter construct (Fig. 2C). We found that mutant MRE A (CDS mut_A), but not mutant MRE C (CDS_mut C), abolished miR-138-mediated repression of the *MYC* CDS (Fig. 2D). This suggests that MRE A, but not MRE C, is critical for miR-138 regulation of *MYC*. In support of this, we found that MRE A was also identified by two other miRNA target prediction platforms, STarMir (Supplementary Table S3) and miRDB (Supplementary Data File S2).

To recapitulate the regulatory effect of miR-138 on the CDS of its target transcript, we inserted the miR-138 MRE A at the 3′ end of the *RLuc* gene, in-frame before the stop codon (Fig. 2E). Consistently, the overexpression of miR-138 significantly downregulated the relative luciferase activity of MRE A. In addition, we created a fusion *RLuc-MYC* gene by cloning the wild-type (WT) *MYC* CDS or *MYC* CDS with mutated MRE A (mut_A) downstream of and in-frame with the *RLuc* gene (Fig. 2F). We found that the mutation of miR-138 MRE A on the *MYC* CDS diminished miR-138-mediated regulation of the *MYC* CDS. Next, we performed biotinylated miR-138 pulldowns and observed a significant enrichment of the *MYC* transcript (Fig. 2G). Taken together, these data provide support for the regulation of the MYC *CDS* by miR-138 via MRE A.

### MiR-138 regulates MYC target gene expression

**Fig. 3:**
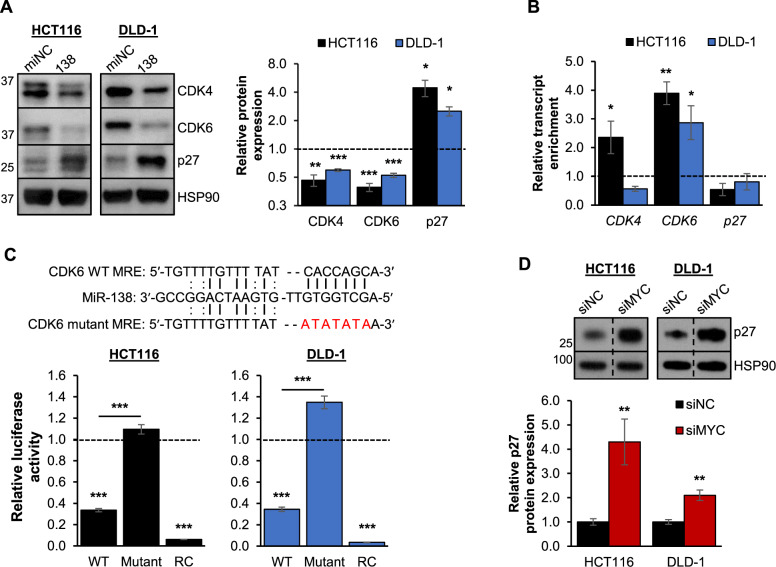
MiR-138 regulates MYC target gene expression. **A** Western blot (left panel) and densitometry quantification (right panel) showing the protein expression of MYC target genes upon the overexpression of miR-138 (138) in HCT116 and DLD-1 cell lines. **B** RT-qPCR analysis of *CDK4*, *CDK6* and *p27* transcript enrichment upon biotinylated miR-138 pulldown in HCT116 and DLD-1 cell lines. **C** The sequences of the wild-type (WT) and mutant miR-138 MREs on the *CDK6* transcript (top panel) and the effect of miR-138 overexpression on the luciferase activity of the MRE-reporter constructs (bottom panel) in HCT116 (left panel) and DLD-1 (right panel) cell lines. RC denotes the reverse complement of miR-138 which was used as positive control. **D** Western blot (top panel) and densitometry quantification (bottom panel) of p27 protein expression upon the knockdown of MYC in HCT116 and DLD-1 cell lines. Mean ± SEM; *N* ≥ 3. *P < 0.05; **P < 0.01; ***P < 0.001.

Next, we explored the effect of miR-138 on MYC target gene expression. MYC has been shown to induce the expression of genes which are involved in cell cycle progression, such as *CDK4* and *CDK6* [21, 22], as well as to repress those involved in cell cycle arrest such as *p27* [23]. Repression of CDK4 and CDK6 levels by miR-138 has been demonstrated in breast cancer cell lines [24]. However, direct regulation was only validated for CDK6 in glioblastoma multiforme [25]. We performed western blot analysis to investigate if these interactions were present in CRC, and observed a significant reduction in CDK4 and CDK6 protein levels upon miR-138 overexpression (Fig. 3A). In addition, we found that the *CDK6* transcript was significantly enriched in the biotinylated miR-138 pulldown in both CRC cell lines, while the *CDK4* transcript was only enriched in HCT116 (Fig. 3B). This suggests that the regulation of *CDK4* by miR-138 might be indirect and/or cell-line specific. Next, we performed MRE-luciferase reporter assays using the validated WT miR-138 MRE on the *CDK6* transcript and a version with mutations in the miR-138 seed region (Fig. 3C). Consistent with other findings, the overexpression of miR-138 decreased the relative luciferase activity of the WT MRE, while this effect was abolished in the mutant (Fig. 3C) [25]. Overall, our data confirmed that *CDK6* was also a direct target of miR-138 in CRC.

Based on in-silico target identification, we identified and validated *LYPLA1* as another direct target of miR-138 in CRC (Supplementary Fig. S2a-f). We also confirmed the growth-promoting role of LYPLA1 in CRC (Supplementary Fig. S2g), which was consistent with its previously described role in non-small cell lung cancer (NSCLC) progression [26].

In addition, miR-138 overexpression increased p27 protein expression (Fig. 3A), but its transcript was not enriched in the biotinylated miR-138 pulldown (Fig. 3B). Further examination showed that the expression of p27 was significantly upregulated upon MYC knockdown in both HCT116 and DLD-1 cells, suggesting that miR-138 exerts an indirect effect on p27 expression via MYC (Fig. 3D). Taken together, miR-138 may have a strong tumor-suppressive effect in CRC by targeting several transcripts involved in cell cycle progression either directly or indirectly via MYC.

### MiR-138 possesses tumor-suppressive properties in CRC

**Fig. 4:**
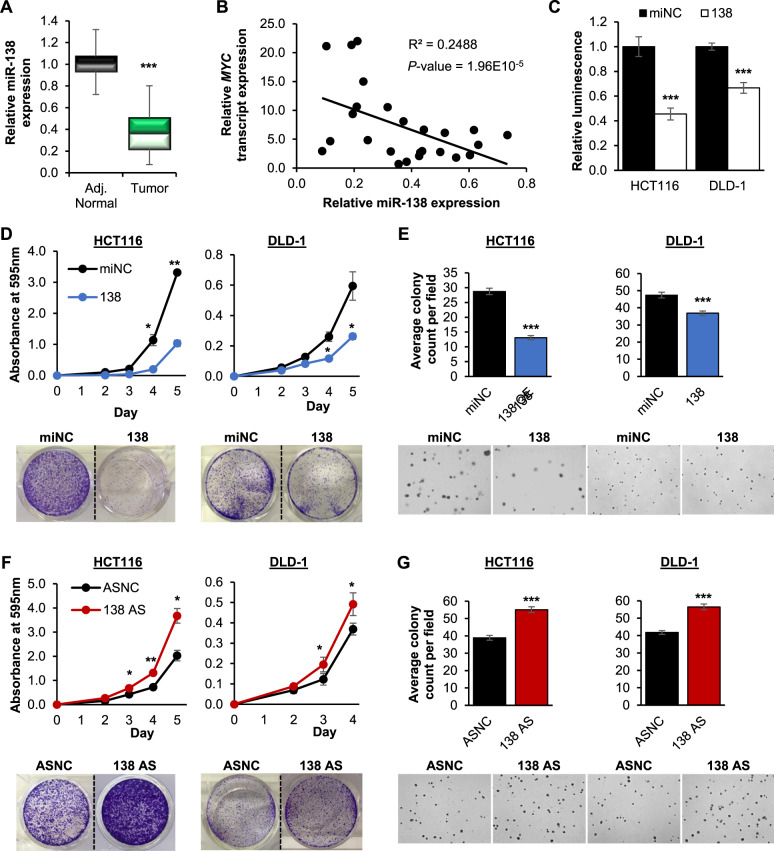
MiR-138 possesses tumor-suppressive properties in CRC. **A** RT-qPCR quantification of miR-138 expression in tumor and adjacent normal samples from CRC patients (*N* = 25). **B** Correlation between miR-138 and *MYC* transcript expression in tumor samples of CRC patients (*N* = 25). **C** CellTiter-Glo^®^ assay examining the effect of miR-138 overexpression on the cell viability of HCT116 and DLD-1 cells. **D, E** Effect of miR-138 overexpression (138) on anchorage-dependent (**D**) and anchorage-independent (**E**) growth of HCT116 (left panel) and DLD-1 (right panel) cells. **F, G** Effect of miR-138 inhibition (138 AS) on anchorage-dependent (**F**) and anchorage-independent growth (**G**) of HCT116 (left panel) and DLD-1 (right panel) cells. The representative images of anchorage-dependent and -independent growth are shown at the bottom panels. **C**, **E** and **G** Mean ± SEM; *N* ≥ 3. **D** and **F** Mean ± STD; *N* ≥ 3. *P < 0.05; **P < 0.01; ***P < 0.001.

The decreased expression of miR-138 in CRC tissues compared to normal colon has been described previously and was found to be associated with a lower degree of malignancy and better survival of CRC patients [27]. Consistent with this data, we found that miR-138 was downregulated in the CRC tumors of our cohort compared to the adjacent normal tissue (Fig. 4A). Critically, there was a significant negative correlation between miR-138 and *MYC* transcript expression in these patients (Fig. 4B). Taken together with our earlier data (Fig. 1C–E), these findings suggest that the downregulation of miR-138 might contribute to the elevated MYC expression in CRC patients.

Upregulation of miR-138 significantly decreased the cell viability of both CRC cell lines (Fig. 4C). In addition, the overexpression of miR-138 decreased (Fig. 4D and E), while its inhibition increased (Fig. 4F and G) both anchorage-dependent and -independent growth of CRC cells. These observations are consistent with several previous reports, and collectively suggest that miR-138 has potent tumor-suppressive properties in CRC [28–30].

### MiR-138 consistently inhibits MYC expression and cell proliferation in other cancers

**Fig. 5:**
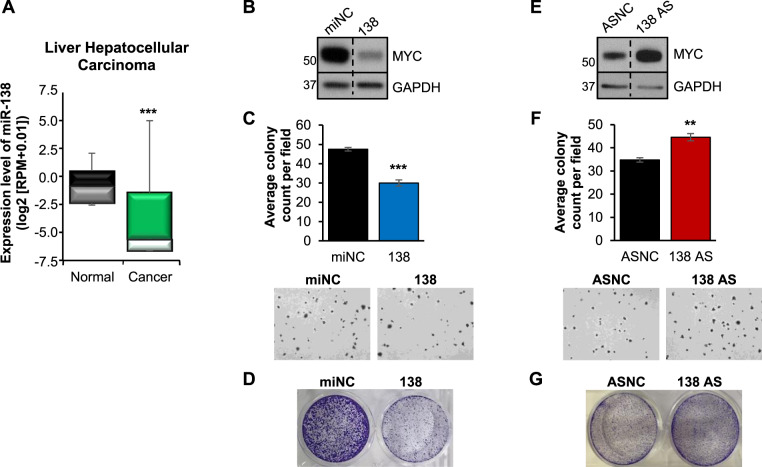
MiR-138 downregulates MYC expression and inhibits growth in liver cancer. **A** MiR-138 expression in hepatocellular carcinoma (HCC) [50 normal versus 370 cancer] based on the TCGA dataset) [33]. **B, C** Effect of miR-138 overexpression (138) on MYC protein level (**B**) and the anchorage-independent growth (**C**) of HepG2 cells. Representative images of the anchorage-independent growth are shown at the bottom panel. **D** Representative image of anchorage-dependent growth of HepG2 cells upon miR-138 overexpression (138). **E, F** Effect of miR-138 inhibition (138 AS) on MYC protein level (**E**) and the anchorage-independent growth (**F**) of HepG2 cells. Representative images of the anchorage-independent growth are shown at the bottom panel. **G** Representative image of anchorage-dependent growth of HepG2 cells upon miR-138 inhibition (138 AS). Mean ± SEM; *N* ≥ 3. **P < 0.01; ***P < 0.001.

MicroRNA regulation can be highly context-specific, and miR-138 has been shown to exhibit oncogenic properties in other cancers such as malignant glioma [31] and triple-negative breast cancer [32]. Thus, we next explored whether miR-138-mediated regulation of MYC extended to other cancers. Based on TCGA expression data, we selected liver HCC, kidney clear cell renal cell carcinoma and lung squamous cell carcinoma for further study as miR-138 was significantly downregulated in these cancers (Fig. 5A and Supplementary Fig. S3a) [33]. In accordance with our data in CRC cell lines, overexpression of miR-138 consistently downregulated MYC expression (Fig. 5B and Supplementary Fig. S3b) and in vitro cell growth (Fig. 5C, D and Supplementary Fig. S3c and S3d) of all three cancer types.

### MiR-138 suppresses MYC-driven carcinogenesis in vivo

The potent repressive effect of miR-138 on MYC expression in cancer cell lines led us to hypothesize that it may also have a significant effect on MYC-driven carcinogenesis in vivo. Of the four cancer types studied in vitro, we focused on liver cancer, which has frequent overexpression of MYC (Supplementary Fig. S3e) without genetic amplification [9, 10]. In addition, *MYC* is a well-established driver of liver cancer initiation and progression, so the identification of novel MYC-regulatory pathways in this cancer may have profound scientific and clinical impact [34, 35]. Further in vitro analyses also showed that MYC expression (Fig. 5E and Supplementary Fig. S3f) and cell growth (Fig. 5F, G and Supplementary Fig. S3g) were upregulated upon miR-138 inhibition in HCC cell lines.

**Fig. 6:**
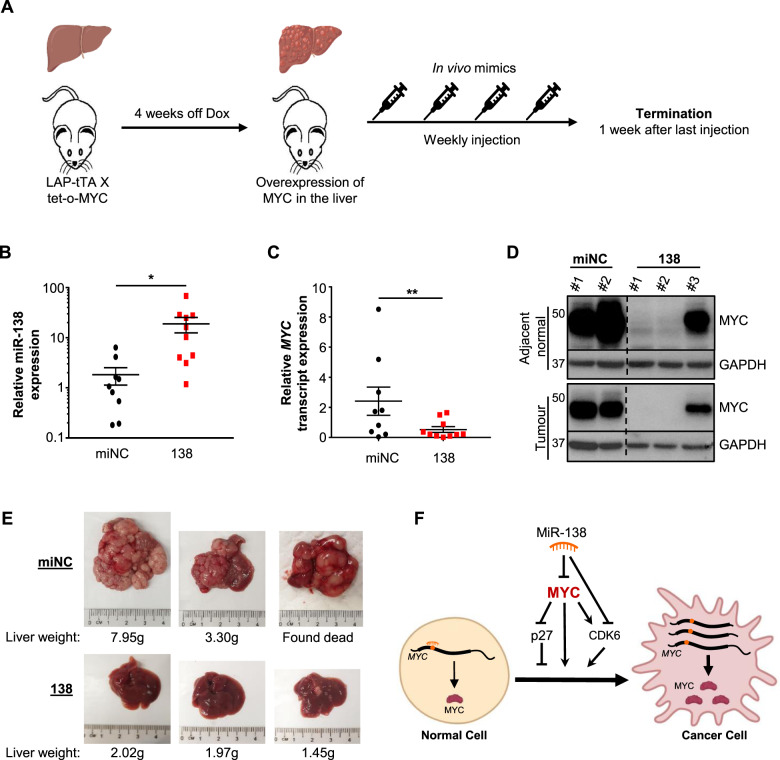
MiR-138 suppresses MYC-driven carcinogenesis in vivo. **A** Schematic showing the timeline of the intravenous miR-138 mimic injection into transgenic mice. **B, C** RT-qPCR analyses of miR-138 (**B**) and *MYC* transcript (**C**) expression in the liver upon miNC and miR-138 mimic injection [9 miNC versus 10 miR-138 mimics injection]. **D** Representative Western blot analysis of MYC protein levels upon miNC and miR-138 mimic injection. **E** Representative images of the mouse livers upon miNC (top panel) and miR-138 (bottom panel) injection. (E – I) Samples were harvested one week after the final mimic injection. **F** Schematic diagram summarizing the effects of miR-138 on MYC and cancer development. Orange circle denotes miR-138 MRE. Mean ± SEM. *P < 0.05; **P < 0.01.

To investigate the growth inhibitory effect of miR-138 in vivo, we performed weekly intravenous miR-138 injections in LAP-tTA X tet-o-MYC transgenic mice which overexpresses human *MYC* CDS in the liver and is widely used to study MYC-driven HCC (Fig. 6A) [36]. Mice were sacrificed a week after the last injection. The mice injected with miR-138 mimics had significantly elevated miR-138 levels in their livers (Fig. 6B) and other organs (Supplementary Fig. S4a), and decreased *Myc* transcript expression compared to those injected with the negative control (Supplementary Fig. S4b). Strikingly, the overexpression of miR-138 in the LAP-tTA X tet-o-MYC mice effectively reduced both *MYC* transcript and protein levels (Fig. 6C, D and Supplementary Fig. S4c), as well as liver tumor growth compared to the negative control (miNC) (Fig. 6E). Collectively, our results highlight the potential utility of miR-138 as a previously uncharacterized inhibitor of MYC-driven carcinogenesis in vivo.

## Discussion

Although widespread 3′UTR shortening has been shown to result in the loss of miRNA targeting, and associated disruption of competing endogenous RNA networks, little is known about the specific 3′UTR populations of key cancer driver genes [37, 38]. In this study, we describe the shortening of the *MYC* 3′UTR in CRC, particularly in the more advanced disease (Fig. 1A). This finding suggests that many of the known *MYC:*miRNA interactions may not be present in certain cancer cells and highlights the need to expand the conventional focus on miRNA target sites in 3′UTRs to include 5′UTRs and coding regions, to map the full landscape of miRNA:target interactions in both normal and cancer cells. This will in turn facilitate the selection of the most promising miRNA:target interactions for the development of potential clinical applications in a disease-specific context. In addition, as 3′UTRs are targeted by other regulators such as RNA-binding proteins (RBPs), their shortening may also lead to the loss of RBP-mediated regulation [39]. Future studies will be key to unravelling the impact of 3′UTR shortening and the concomitant loss of these RNA:RNA and RNA:protein interactions on the expression and function of key cancer genes [38].

The discovery of the heterogenous *MYC* transcript population in cancer cells led us to focus on the identification of miRNAs, which target the *MYC* CDS as this is present in all *MYC* transcript variants. We describe a previously uncharacterized role for the tumor-suppressive miR-138 as a potent regulator of MYC expression in both CRC and HCC (Fig. 6F). The combination of *MYC* 3′UTR shortening and the downregulation of miR-138 may contribute to increased MYC expression in cancer cells. Low levels of miR-138 also directly or indirectly affect the expression of MYC target genes such as *CDK6* and *p27*. Cumulatively, these effects promote uncontrolled cell proliferation and subsequent cancer development.

Critically, we show that treatment with miR-138 mimics was able to reduce MYC expression and tumor growth in a well-established transgenic mouse model for MYC-driven HCC. However, as miR-138 has been shown to exhibit context-specific tumor suppressive or oncogenic properties, it is important to examine its potential delivery into tissues in which it acts as an oncogenic miRNA. For example, miR-138 has been validated as a pro-survival oncogenic miRNA in malignant glioma [31]. In our experiments, we did not observe significant enrichment of miR-138 in the brain upon mimic injection (Supplementary Fig. S4a). This is likely to be due to the blood-brain barrier, which diminishes the risk of malignant glioma development upon mimic administration. Interestingly, there was a moderate level of miR-138 enrichment in the kidney, suggesting that the mimics may be readily eliminated from the body hence minimizing potential chronic toxicity.

In recent years, there has been remarkable progress in the development of miRNAs as diagnostic and prognostic tools as well as therapeutic targets [40, 41]. In addition, novel delivery systems such as viral vectors [42, 43], lipid-based nanocarriers [44, 45], cell-derived membrane vesicles [46, 47], polymeric vectors [48–50], as well as 3D scaffold-based delivery systems [51] have significantly improved the delivery efficiency of both antisense miRNAs (antimiRs) and miRNA mimics to suppress or replenish miRNAs, respectively [13, 52]. Some studies which have progressed into clinical trials for cancer treatment include that of miR-16 mimics (MesomiR1) (ClinicalTrials.gov identifier: NCT02369198) for the treatment of mesothelioma and NSCLC which is currently in Phase I clinical trials [53], and antimiR-155 (MRG-106) (ClinicalTrials.gov identifier: NCT02580552) for the treatment of T cell lymphoma and mycosis fungoides in Phase II clinical trials [54].

Despite these progressive breakthroughs in miRNA research, significant challenges remain before we can successfully translate them into clinical therapeutics. Firstly, as miRNAs may have different targets in different tissues and organs, it is critical to deliver the miRNA therapeutic specifically to the targeted tissue to avoid unintended side effects [55]. In addition, diverse epigenetic, transcriptional and hormonal mechanisms may underlie miRNA regulation, increasing the complexity of their therapeutic application [55]. Therefore, deeper insights into miRNA targeting patterns and efficient delivery in a tissue-specific manner will be key to unlocking the potential of miRNA therapeutics while minimizing off-target effects and the associated risks.

We anticipate that miRNA therapy will blossom into an integral part of the precision medicine toolbox in the future, and hope that our identification of the miR-138:MYC interaction may launch new avenues for the targeting of the heterogeneous MYC transcript population and the subsequent treatment of MYC-driven cancers.

## Materials and methods

### 3′ rapid amplification of cDNA ends (3′ RACE)

The 3′ RACE was performed using the RLM-RACE Kit (FirstChoice^®^) following the manufacturer’s protocol. 1 µg of total RNA from adjacent normal and tumor samples of CRC patients was used for the experiment. Primers used for the 3′ RACE are listed in Supplementary Table S4. The amplified complementary DNAs (cDNAs) were cloned into pcDNA4-TO-Puromycin-mVenus-MAP. Sanger sequencing was performed on ten clones from each sample. The clones were analyzed for the length of the *MYC* 3′UTR based on a few criteria; the presence of (1) both the forward and reverse inner PCR primers, (2) both the HindIII and BamHI restriction sites, and (3) the *MYC* CDS and stop codon preceding the 3′UTR. The poly(A) at the end of the 3′UTR was included in the calculation of the 3′UTR length.

### Clinical samples

CRC and adjacent normal tissues were obtained from the National University Hospital, Singapore. This study was approved by the relevant Institutional Review Boards in Singapore, and informed consent was obtained from all subjects. 10–20 mg of fresh tissue was homogenized using a pestle plastic homogenizer and passed through a 21-gauge needle fitted to an RNase-free syringe. DNA, RNA, and protein were extracted using the QIAGEN AllPrep DNA/RNA/Protein Mini Kit according to the manufacturer’s protocol.

### Reagents

Reagents are as follows: anti-GAPDH (sc-47724), anti-HSP90 (sc-69703) and anti-YFP (sc-32897) antibodies (Santa Cruz Biotechnology); anti-MYC (ab32072) antibody (Abcam); anti-LYPLA1 (GTX104398) antibody (GeneTex); anti-p27 (3686S), anti-CDK4 (12790S) and anti-CDK6 (3136S) antibodies (Cell Signaling Technology (CST)); Rabbit IgG (301-001-003) antibody (Jackson ImmunoResearch Laboratories); miRIDIAN miRNA mimics for non-targeting control (NC) (miNC) and miRNAs (138, 139, 28, 99b, 193a, 197, 125a, 503, 744), miRIDIAN miRNA inhibitors for NC (ASNC), miR-28-5p (28 AS), miR-138 (138 AS), and miR-139-3p (139 AS), biotinylated miRNA reagents (NC and 138), siGENOME siRNA for NC (siNC) and MYC (siMYC), DharmaFECT 1 (Dharmacon), TRIZOL^®^, Lipofectamine 3000, Dulbecco’s Modified Eagle Medium (DMEM), Roswell Park Memorial Institute (RPMI) 1640 medium, Opti-MEM^™^ reduced serum media, fetal bovine serum (FBS), Dynabeads^™^ M-280 Streptavidin (ThermoFisher Scientific), psiCHECK-2 (Promega), pcDNA4-Puro (Addgene).

### Cell culture and transfection

HCT116, HepG2, and Calu-1 cells were maintained in DMEM, while DLD-1, SNU-398 and 786-O cells were maintained in RPMI, supplemented with 10% FBS, 1% penicillin/streptomycin and 1% L-glutamine. Both cell lines were grown at 37 °C in a humidified atmosphere with 5% CO_2_. HCT116 and DLD-1 cell lines were obtained from Horizon Discovery, 786-O, HepG2 and SNU-398 cell lines were obtained from ATCC, while Calu-1 cell line was a kind gift from Prof. Goh Boon Cher (Cancer Science Institute of Singapore). Mycoplasma contamination test was performed, and the results were negative. Cells were transfected with 50 nM of miRNA mimics, inhibitors or siRNAs using DharmaFECT 1 as per the manufacturer’s protocol at a seeding density of 150,000 cells per well in 12-well dishes. For miRNA inhibitors transfection, the cells were transfected in serum-free medium for 48 h.

### MS2-tagged RNA affinity purification (MS2- TRAP) pulldown

HCT116 cells were seeded at a density of one million cells per well in six-well plates. Cells were co-transfected with plasmids containing MS2-binding protein (MS2-BP) fused with the yellow fluorescent protein (YFP) and the *MYC* CDS tagged with the MS2 hairpin sequence. The protocol was taken from the RIP-ChIP protocol [56].

### MicroRNA target prediction and databases

MicroRNA target prediction was performed using RNA22 which is available at https://cm.jefferson.edu/rna22/Interactive/ [20]. Prediction of miR-138 MREs on the human *MYC* CDS was also performed using STarMir, which is available at https://sfold.wadsworth.org/cgi-bin/starmirtest2.pl and miRDB, which is available at http://mirdb.org/. MiR-138 targets were obtained from ENCORI (The Encyclopedia of RNA Interactomes), which is available at http://starbase.sysu.edu.cn/index.php [33]. The miR-138 targets were predicted by more than 4 prediction platforms and were downregulated in CRC and HCC.

### Protein extraction and western blot analysis

Cells were trypsinized and harvested in the appropriate growth medium and lysed on ice for 10 min in protein lysis buffer (PLB) (10 mM HEPES pH 7.0, 0.1 M KCl, 5 mM MgCl_2_, 25 mM EDTA pH 8.0, 0.5% (v/v) NP-40, Proteinase Inhibitor, 20 µM DTT), followed by centrifugation at 16,000 x *g* for 15 min. The concentrations of the lysates were measured using Bradford Protein Assay (Bio-Rad). 8–12 µg of protein lysates was separated on 4–12% Bis-Tris NuPAGE^®^ Precast gels (ThermoFisher Scientific) and transferred to PVDF membranes using the Mini Trans-Blot^®^ Electrophoretic Transfer Cell (BioRad) in transfer buffer (2.5 mM Tris, 19.2 mM glycine and 10% (v/v) methanol). The membranes were probed with specific primary antibodies followed by the corresponding secondary antibodies.

### RNA extraction, real-time quantitative PCR (RT-qPCR)

Total RNA was isolated using the TRIZOL^®^ reagent and the PureLink^®^ RNA Mini Kit (Ambion) as per the manufacturer’s protocol. 500 ng of total RNA was used to synthesize the cDNA using the High-Capacity cDNA Reverse Transcription Kit (ThermoFisher Scientific). qPCR was performed using the PowerUp^TM^ SYBR^®^ Green Master Mix (Applied Biosystems) on the QuantStudio 5 Real-Time PCR System. The primers used for RT-qPCR are listed in Supplementary Table S5.

### Plasmids

Oligonucleotides for the MREs (Supplementary Table S6) were annealed by mixing 100 µM of each primer in 5× sequencing and annealing buffer (1 M Tris HCl pH 7.5, 5 M NaCl, 1 M MgCl_2_). The mixture was heated at 95 °C for 5 min and cooled to room temperature and ligated into the digested psiCHECK-2 vector.

### Luciferase reporter assays

For dual-luciferase reporter assays, cells were seeded at a density of 50,000 cells per well in 24-well plates a day prior to transfection. 5 ng of psiCHECK-2 plasmids was co-transfected with 50 nM miRNA mimics. At 48 h post-transfection, cells were washed in PBS, lysed and luminescence was measured following the manufacturer’s instructions (Promega).

### Site-directed mutagenesis (SDM)

SDM was performed using the QuikChange Lightning Multi Site-Directed Mutagenesis Kit (Agilent Technologies) following the manufacturer’s protocol. SDM primers are listed in Supplementary Table S7.

### Biotinylated MiRNA pulldown

HCT116 and DLD-1 cells were seeded at a density of 500,000 cells per well in six-well plates. Cells were transfected with biotinylated miRNA mimics (Dharmacon), for 48 h prior to harvesting in protein lysis buffer. The pulldown experiment was performed as previously described [57]. RNAs bound to the miRNA baits were harvested using Trizol LS and purified using phenol: chloroform: isoamyl (25:24:1), followed by chloroform: isoamyl (24:1) (Sigma-Aldrich).

### CellTiter-Glo^®^ luminescent cell viability

Cells were transfected with 50 nM of miRNA mimics and seeded at a density of 1000 cells per well in 96-well plates. After 48 h transfection, 50 μl of CellTiter-Glo^®^ assay reagents (Promega) were added to each well and incubated for 5 min at room temperature before measuring the luminescence readings.

### Anchorage-dependent growth assay

At 24 h of post-transfection, cells were split to five wells at 30,000 cells per well density in 12-well plates. Cells were fixed with 10% neutral buffered formalin solution (Sigma-Aldrich) from day 0 to day 5 (excluding day 1). On day 5, cells were stained with 0.5% crystal violet (Sigma-Aldrich) for 5 min. The excess stain was removed by rinsing with water. After 2 h of drying, the crystal violet stain was dissolved in 10% acetic acid (Sigma-Aldrich). The plates were incubated at room temperature for 1 h with shaking. The absorbance was measured at 595 nm.

### Soft agar assay

A 0.6% agarose base was prepared in six-well plates. 24 h after transfection, cells were trypsinized, resuspended and diluted to 15,000 cells per well in growth medium. The cells were mixed with agarose to obtain a 0.3% top layer agar which was added above the 0.6% base agar. The cells were maintained at 37 °C and the growth medium was changed twice a week. Images were taken at ×4 magnification once every 5 days up to 14 days and quantified using ImageJ.

### Transgenic mice

Tet-o-MYC transgenic mice (JAX stock #019376) [58] and LAP-tTA mice (JAX stock #003563) [59] were purchased from The Jackson Laboratory. The two mouse strains were crossed to produce transgenic mice which carry both the tet-o-MYC and LAP-tTA genes [36]. To induce *MYC* transgene expression, the diet containing doxycycline was replaced with normal chow diet when mice were 4–5 weeks old. Male and female mice with both transgenes overexpressed human *MYC* CDS in the liver were used in this study. All animal works were approved by NUS Institutional Animal Care and Use Committee (IACUC), protocol number R19-0852.

### In vivo MiRNA mimics tail-vein injection

mirVana^™^ miRNA mimics, Negative Control #1 (miNC) and mirVana^®^ miRNA mimic - hsa-miR-138-5p (138), were purchased from ThermoFisher Scientific. Invivofectamine^TM^ 3.0 reagent (ThermoFisher Scientific) was used for the delivery of 20 µg of miRNA mimic per mouse, following the manufacturer’s protocol [60, 61]. Five mice were randomly allocated into each miNC or 138 group for each experimental set. The miRNA mimics were delivered intravenously into the mice every week with a total of four doses by the end of the experiment. One week after the final injection, the mice were sacrificed, and the organs were harvested for downstream biochemical analyses. Experiments, data collection and analysis were performed by three investigators, with at least one blinded investigator.

### Statistical analysis

All in vitro experiments were performed three times independently. Sample size was chosen based on ${{{{{\mathrm{N = }}}}}}\frac{{\log \beta }}{{\log p}}$ whereby *β* is 0.05 and *p* is 0.5, thus the sample size was 4.32 (round up to 5) [62]. Values calculated from multiple independent experiments were presented as mean ± SEM, while data shown from a representative experiment were presented as mean ± SD. Unpaired Student’s *t* test was used to analyze the statistical significance whereby *P* values < 0.05 were considered statistically significant. *,*P* < 0.05; **,*P* < 0.01; ***,*P* < 0.001.

## Supplementary information

Supplementary Figures

Supplementary Tables

Supplementary Data File S1

Supplementary Data File S2
