# Supplementary material for: MiR-138 is a potent regulator of the heterogenous MYC transcript population in cancers
Source: Oncogene. 2021 Dec 22;41(8):1178–89. doi: 10.1038/s41388-021-02084-x (PMC8856960; doi:10.1038/s41388-021-02084-x)
Supplement: Supplementary file 1 — Supplementary Figures [file 41388_2021_2084_MOESM1_ESM.pdf]

## Supplementary Fig. S1

A

| MicroRNAs | Relative Enrichment | |
|---|---|---|
| | Set A | Set B |
| MiR-193a-5p | 1.61 | 1.54 |
| MiR-197-3p | 1.74 | 1.86 |
| MiR-28-5p | 26.35 | 1.97 |
| MiR-99b-3p | 1.62 | 1.51 |

B

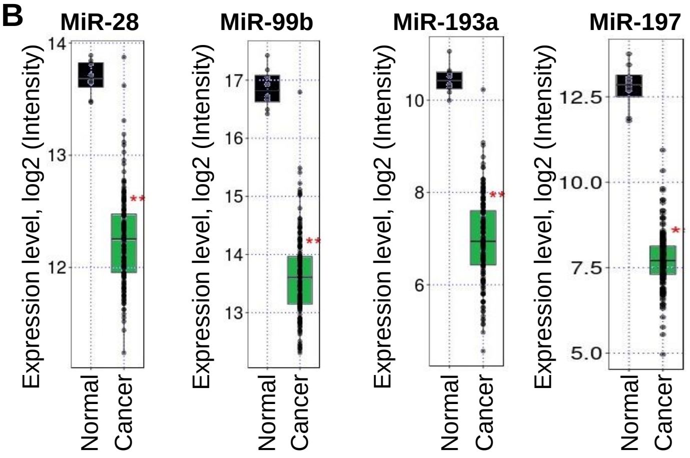


C

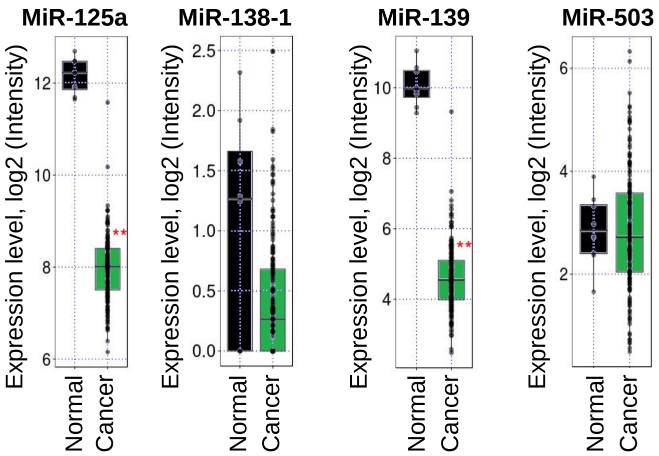


D

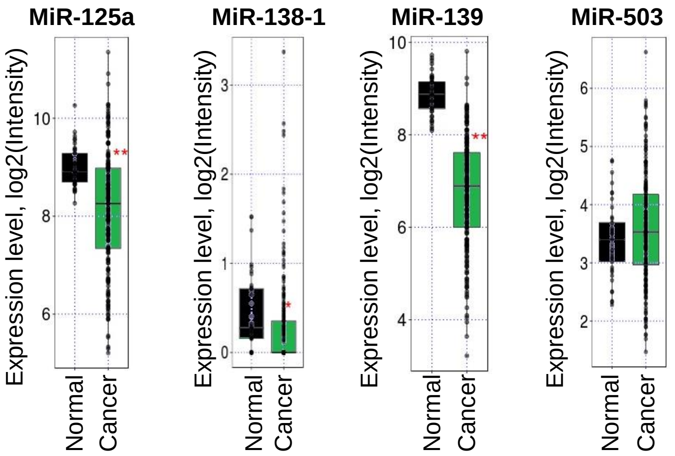


E

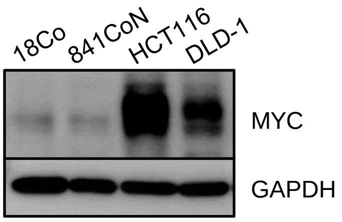


F

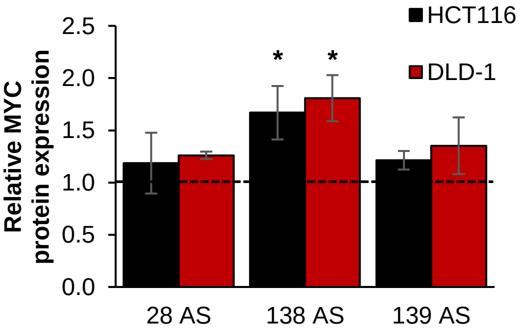


**Supplementary Fig. S1 Identification of potential *MYC* CDS-targeting miRNAs.**

**a** Enrichment of candidate miRNAs in the MS2-TRAP pulldown of the *MYC* CDS relative to EV control. **b** Expression of miRNAs (miR-28-5p, miR-99b-3p, miR-193a-5p, and miR-197-3p) enriched in the *MYC* CDS pulldown in the TCGA-COAD dataset [8 normal versus 178 cancer]. **c, d** Expression of predicted *MYC* CDS-targeting miRNAs (miR-125a-3p, miR-138, miR-139-3p and miR-503-5p) in the TCGA-COAD [8 normal versus 178 cancer] (**c**) and TCGA-LIHC [41 normal versus 210 cancer] (**d**) datasets. **e** Western blot analysis of MYC protein expression in normal colon (CCD-18Co and CCD841CoN) and CRC cell lines (HCT116 and DLD-1). **f** Densitometry quantification of MYC protein expression upon miRNA inhibition by antisense miRNA inhibitors (AS) in HCT116 and DLD-1. Mean ± SEM; N ≥ 3. *$P < 0.05$; **$P < 0.01$; ***$P < 0.001$.

## Supplementary Fig. S2

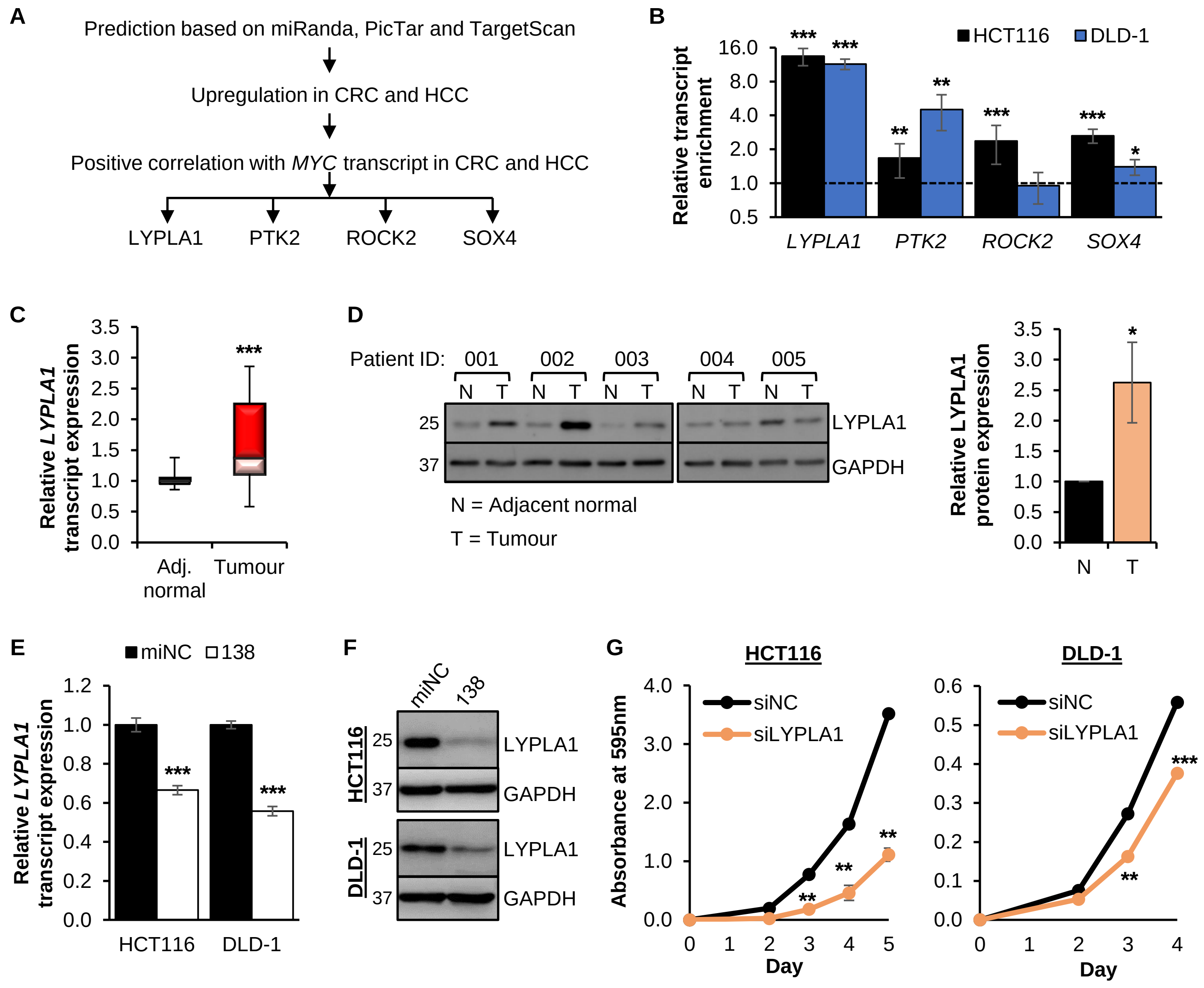


**Supplementary Fig S2. MiR-138-mediated regulation of LYPLA1**.

**a** Flowchart outlining the steps for the identification of potential miR-138 targets in CRC and HCC. The shortlisted targets were predicted by all three platforms. **b** RT-qPCR analysis of potential miR-138 target transcript enrichment upon biotinylated miR-138 pulldown in HCT116 and DLD-1 cell lines. **c, d** RT-qPCR and western blot analyses of *LYPLA1* transcript (**c**) and protein (**d**) levels in adjacent normal (N) and tumor (T) samples from CRC patients. **e, f** Effect of miR-138 overexpression on the transcript and protein expression of *LYPLA1* detected by RT-qPCR (**e**) and western blot (**f**) analyses respectively in HCT116 and DLD-1. **g** Effect of LYPLA1 knockdown on anchorage-dependent growth of HCT116 (left panel) and DLD-1 (right panel). (**b**, **c**, **d** and **e**) Mean ± SEM; N ≥ 3. (**g**) Mean ± STD; N ≥ 3. **P* < 0.05; ***P* < 0.01; ****P* < 0.001.

## Supplementary Fig. S3

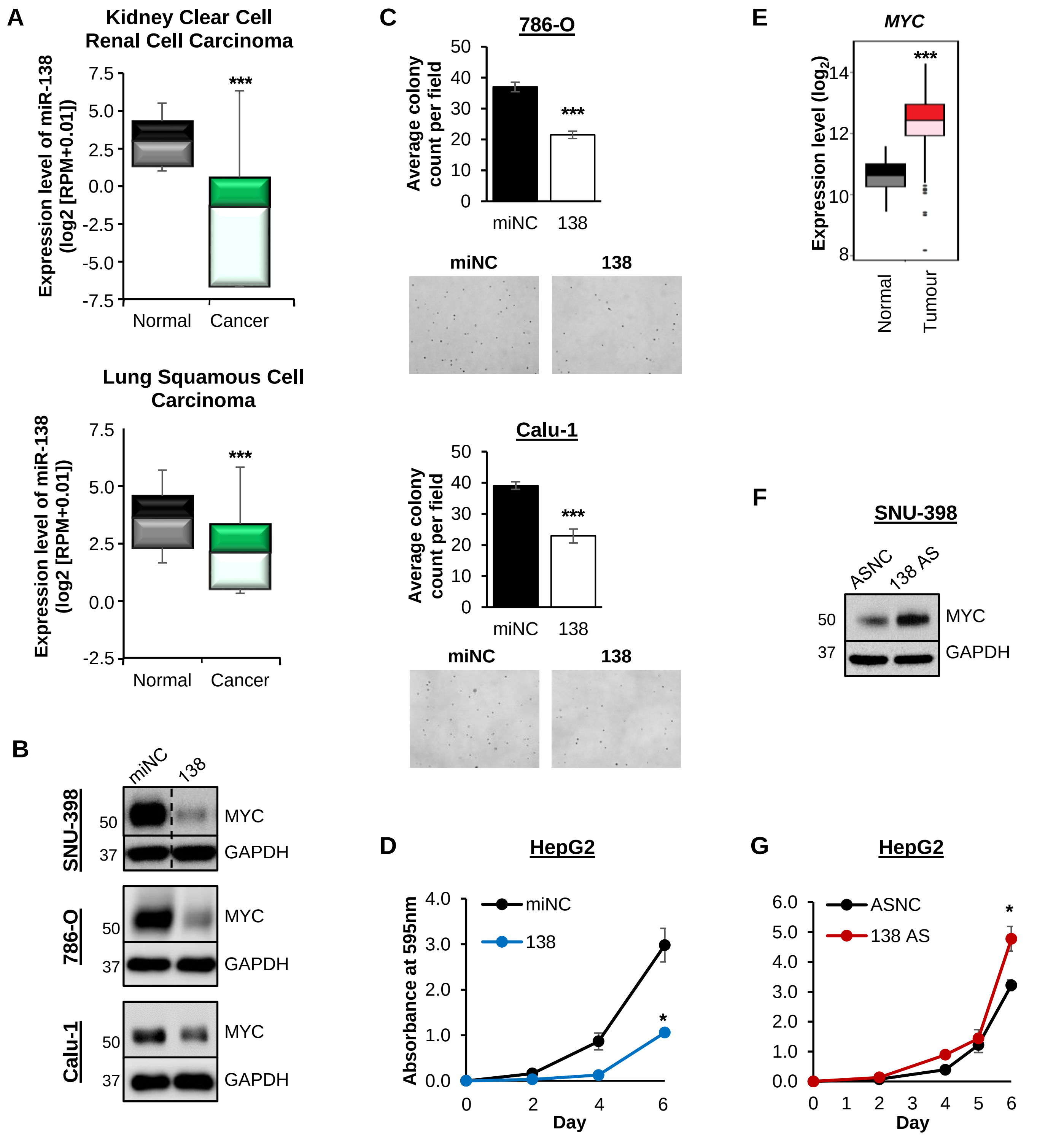


**Supplementary Fig S3. MiR-138 consistently inhibits MYC expression and cell proliferation in other cancers.**

**a** MiR-138 expression in the kidney clear cell renal cell carcinoma (ccRCC) [71 normal versus 517 cancers] (top panel), and lung squamous cell carcinoma (SCC) [38 normal versus 475 cancers] (bottom panel) based on the TCGA datasets. **b** Effect of miR-138 expression (138) on MYC protein expression in liver hepatocellular carcinoma (HCC) cell line, SNU-398 (top panel), kidney ccRCC cell line, 786-O (middle panel), and lung SCC cell line, Calu-1 (bottom panel). **c, d** Effects of miR-138 overexpression (138) on the anchorage-independent growth 786-O (top panel), and Calu-1 (bottom panel) (**c**) and anchorage-dependent growth of HepG2 (**d**). **e** Analysis of *MYC* transcript expression in the TCGA-LIHC dataset [41 normal versus 210 cancer]. **f** Western blot analysis of MYC protein expression upon miR-138 inhibition (138 AS) in the HCC cell line, SNU-398. **g** Effect of miR-138 inhibition (138 AS) on the anchorage-dependent growth of HepG2. (**c**) Mean ± SEM; N ≥ 3. (**d** and **g**) Mean ± STD; N ≥ 3. $^{*}P < 0.05$; $^{***}P < 0.001$.

## Supplementary Fig. S4

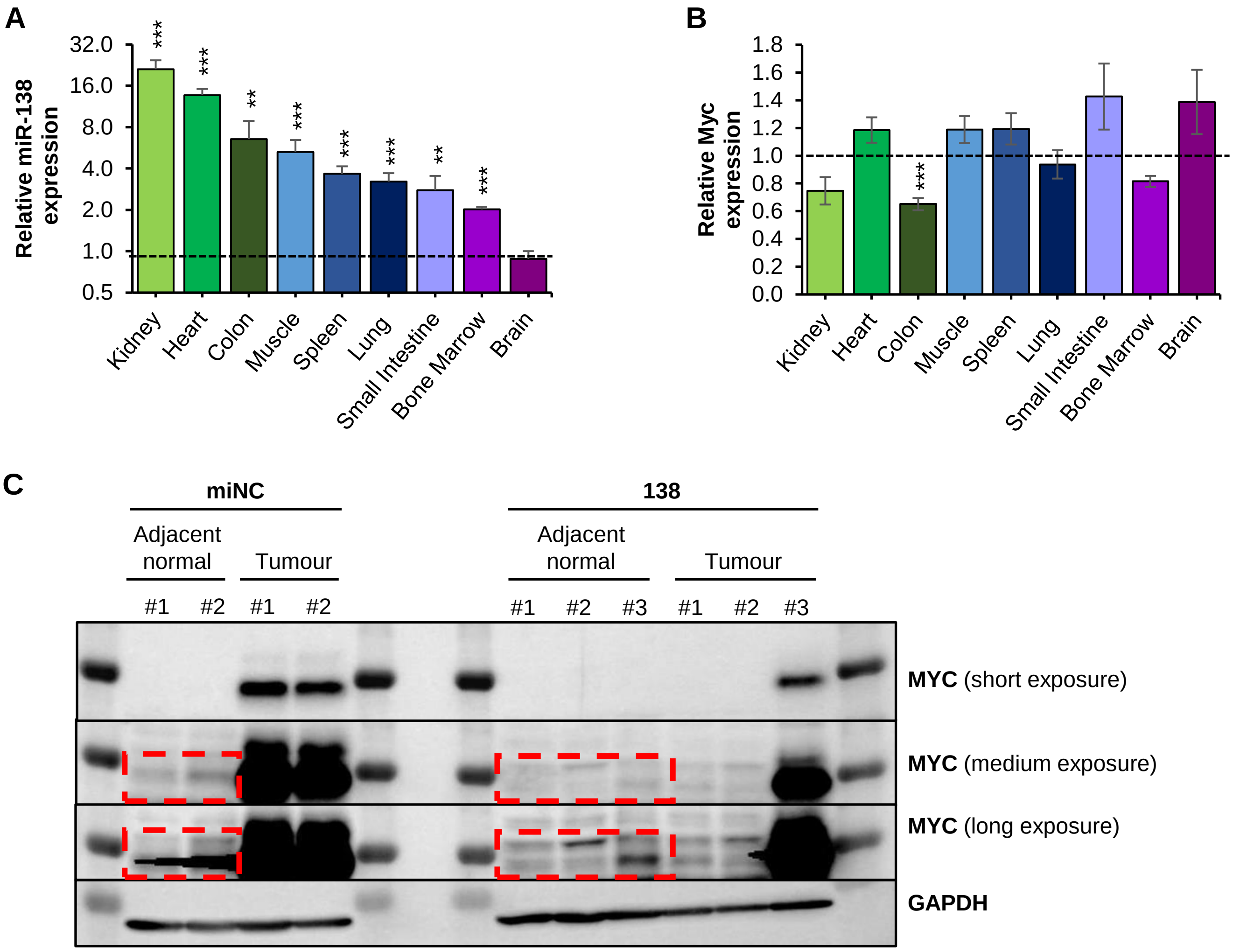


**Supplementary Fig S4. Effect of miR-138 on MYC-driven carcinogenesis in vivo.**

**a, b** RT-qPCR analyses of miR-138 (**a**) and Myc transcript (**b**) expression in different tissues after the miR-138 mimic injection compared to miNC injection (N = 5). **c** Western blot analysis of MYC protein levels in LAP-tTA x tet-o-MYC mice one week after the last injection of miNC or miR-138 mimics. The red boxes highlight MYC protein expression in the adjacent normal samples. Mean ± SEM. $^{**}P < 0.01$; $^{***}P < 0.001$.
