# Supplementary material for: MiR-138 is a potent regulator of the heterogenous MYC transcript population in cancers
Source: Oncogene. 2021 Dec 22;41(8):1178–89. doi: 10.1038/s41388-021-02084-x (PMC8856960; doi:10.1038/s41388-021-02084-x)
Supplement: Supplementary file 2 — Supplementary Tables [file 41388_2021_2084_MOESM2_ESM.pdf]

Supplementary Tables

Supplementary Table S1: Predicted miRNA binding sites on the human and mouse *MYC* CDS by RNA22

<table>
<tr><th>MicroRNAs</th><th>Heteroduplex in human</th><th>Heteroduplex in mouse</th><th>Position in human/mouse</th></tr>
<tr><td rowspan="3">MiR-125a-3p</td><td>TGCTGCCCA--CCCCGCCCCTGT
 ||| |||| || | |||||
CCGA-GGGTTCTTGGAGTGGACA</td><td>TGCTTCCCA--CCCCGCCCCTGT
 ||| |||| || | |||||
CCGA-GGGTTCTTGGAGTGGACA</td><td>164/164</td></tr>
<tr><td>GGTGAACCA-GAGTTTCATCTGC
||: ||| ||: |||:|||
CCG-AGGGTTCTTGGAGTGGACA</td><td>GGTGAACCA-GAGCTTCATCTGC
||: ||| ||:|:|||:|||
CCG-AGGGTTCTTGGAGTGGACA</td><td>330/333</td></tr>
<tr><td>ACCTCCTCACAGCC-CACTGGT
  ||||: | |:|| |||: ||
CCGAGGGTTCTTGGAGTGGACA</td><td>ACCTCCGCA-CAGCC-CACTGGT
  |||| || |:|| |||: ||
CCGAGG-GTTCTTGGAGTGGACA</td><td>867/867</td></tr>
<tr><td rowspan="3">MiR-138-5p</td><td>AGG-AGGA-GAACTTCTACCAGCA
 || || || :||||||
GCCGGACTAAGTG-TTGTGGTCGA</td><td>AGGAAGAGAATTTC-TATCACCAGCA
 || || ||| |||||||
GCC-GGACT--AAGTGTTGTGGTCGA</td><td>80/80</td></tr>
<tr><td>GCGTCTG-CTC-CACCTCCAGCT
  |:||| || || | ||||||
GCCGGACTAAGTGTTGTGGTCGA</td><td>GCGTCTG-CTC-CACCTCCAGCC
  |:||| || || | |||||
GCCGGACTAAGTGTTGTGGTCGA</td><td>506/509</td></tr>
<tr><td>GTGCCACGTCTCCACA-CATCAGCA
  ||| |||| ||:||||
GCCGG--ACTAAGTGTTGTGGTCGA</td><td>GTGCCACGTCTCCAC-TCACCAGCA
  ||| ||| |||||||
GCCGG--ACTAAGTGTTGTGGTCGA</td><td>897/897</td></tr>
<tr><td>MiR-139-3p</td><td>TCCTCAAGAGGTGCCACGTCTCCA
         || ||| ||||||||
TGAGGTTGTCC-CGGCGCAGAGGT</td><td>CCTCAAGAGGTGCCACGTCTCCA
| ||| ||| ||| ||||||||
GAGGTTGTCC-CGGCGCAGAGGT</td><td>887/888</td></tr>
<tr><td>MiR-503-5p</td><td>CTGGAAGAA--ATTCGAGCTGCTG
 || |||| ||| ||||||:
GAC-GTCTTGACAAGGGCGACGAT</td><td>CTGGAAGA-AATTCGAGCTGCTT
||| | | ||| ||||||
GACGTCATGACAAGGGCGACGAT</td><td>147/147</td></tr>
</table>

MiR-744-5p	AGCTG-CTGCCC--ACCCCGCC<br>|||| |||| |||||| ACGACAATCGGGATCGGGGCGT	AGCTG-CTTCCC--ACCCCGCC<br>|||| ||| |||||| ACGACAATCGGGATCGGGGCGT	161/161

For the heteroduplex, the top strand denotes the MRE sequence in the 5′ - 3′ direction, while the bottom strand denotes the miRNA sequence in the 3′ - 5′ direction.

Supplementary Table S2: Predicted miR-138 MREs on the human and mouse *MYC* CDS by RNA22

MREs	Heteroduplex (Human)	Heteroduplex (Mouse)	Leftmost position (Human/Mouse)
A	AGG-AGGA-GAACTTCTACCAGCA<br>|| || || :|||||| GCCGGACTAAGTG-TTGTGGTCGA	AGGAAGAGAATTTC-TATCACCAGCA<br>|| || ||| ||||||| GCC-GGACT--AAGTGTTGTGGTCGA	80/80
B	GCGTCTG-CTC-CACCTCCAGCT<br>|:||| || || | |||||| GCCGGACTAAGTGTTGTGGTCGA	GCGTCTG-CTC-CACCTCCAGCC<br>|:||| || || | ||||| GCCGGACTAAGTGTTGTGGTCGA	506/509
C	GTGCCACGTCTCCACA-CATCAGCA<br>||| |||| ||:|||| GCCGG--ACTAAGTGTTGTGGTCGA	GTGCCACGTCTCCAC-TCACCAGCA<br>||| ||| ||||||| GCCGG--ACTAAGTGTTGTGGTCGA	897/897

For the heteroduplex, the top strand denotes the MRE sequence in the 5′ - 3′ direction, while the bottom strand denotes the miRNA sequence in the 3′ - 5′ direction. Red font indicates the different nucleotides between the human and mouse *MYC* sequences.

Supplementary Table S3: Predicted miR-138 MREs on the human *MYC* CDS by STarMir

<table>
<tr><th>Sites</th><th>Predicted Bindings</th></tr>
<tr><td>1</td><td><pre>
5'->3'   A AG   GA   UUC   A
Target   81   GG   GA   AC   UACCAGC   100
               || || || |||||||
miRNA   23   CC   CU   UG   GUGGUCG   1
3'->5'   G GGA AAG UU   A
</pre></td></tr>
<tr><td>2</td><td><pre>
5'->3'   U   GG GCUUCUC   CG   G
Target   268   GGC GA   CACGGC ACCAGCU   296
               ||| || |||||| |||||||
miRNA   23   CCG CU   GUGUUG UGGUCGA   1
3'->5'   G   GA AA
</pre></td></tr>
<tr><td>3</td><td><pre>
5'->3'   C   ACGA GA CC   CAAA   N
Target   356   CGG   C GA UUCAU   AACAUCA   385
               ||| | || ||||| |||||||
miRNA   23   GCC   G CU AAGUG   UUGUGGU   1
3'->5'   G   A   CGA
</pre></td></tr>
<tr><td>4</td><td><pre>
5'->3'   U U   CAA GGUGC   UCUCCAC   A
Target   885   GG CCU   GA   CACG   ACAUCAGC   919
               || ||| || |||| ||||||||
miRNA   23   CC GGA   CU   GUGU   UGUGGUCG   1
3'->5'   G   AA   A
</pre></td></tr>
</table>

Site 1 is the same site as MRE_A predicted by RNA22 and site 1 predicted by miRDB.

Supplementary Table S4: List of primers for *MYC* 3′ RACE

Primer Names	Primer Sequences (5′-3′)
MYC 3′ RACE outer	GTCCAAGCAGAGGAGCAAAA
MYC 3′ RACE inner	CCCAAGCTTGGGAAGAGGACTTGTTGCGGAAA

Supplementary Table S5: Primers for RT-qPCR

Gene Names	Forward Primers (5′-3′)	Reverse Primers (5′-3′)
MYC	TTCGGGTAGTGGAAAACCAG	CAGCAGCTCGAATTTCTTCC
GAPDH	AGCCACATCGCTCAGACAC	GCCCAATACGACCAAATCC
ROCK2	GAGGTCTACAGATGAAGGCAGA	CCTTGTGACGAACCAACTGC
LYPLA1	GAGATACTGGGCACGGATGG	TGTAACAGGCCTAACAGGCG
PTK2	GTGCTCTTGGTTCAAGCTGGAT	ACTTGAGTGAAGTCAGCAAGATGTGT
SOX4	GGTCTCTAGTTCTTGCACGCTC	CGGAATCGGCACTAAGGAG
P27	ACTCGCCGTGTCAATCATTT	AACACCCCGAAAAGACGAG
CDK4	TTGTCCGGCTGATGGAC	GGGTGCCTTGTCCAGATA
CDK6	CCACTTACTTGGATAAAGTTCCAGA	CAGAATGTTCTGTGGTTTTAGATCG

Supplementary Table S6: Oligonucleotides for cloning miR-138 MREs into psiCHECK-2

Oligo Names	Oligonucleotide Sequences	Restriction Sites
MRE A Forward	TCGAGAGGAGGAGAACTTCTACCAGCAGC	XhoI
MRE A Reverse	GGCCGCTGCTGGTAGAAGTTCTCCTCCTC	NotI
MRE B Forward	TCGAGGCGTCTGCTCCACCTCCAGCTGC	XhoI
MRE B Reverse	GGCCGCAGCTGGAGGTGGAGCAGACGCC	NotI
MRE C Forward	TCGAGGTGCCACGTCTCCACACATCAGCAGC	XhoI
MRE C Reverse	GGCCGCTGCTGATGTGTGGAGACGTGGCACC	NotI
RC Forward	TCGAGCGGCCTGATTCACAACACCAGCTGC	XhoI
RC Reverse	GGCCGCAGCTGGTGTTGTGAATCAGGCCGC	NotI
CDK6 WT Forward	TCGAGCTTGTTTTGTTTTATCACCAGCAGC	XhoI
CDK6 WT Reverse	GGCCGCTGCTGGTGATAAAACAAAACAAGC	NotI
CDK6 Mutant Forward	TCGAGCTTGTTTTGTTTTATATATATATAAGC	XhoI
CDK6 Mutant Reverse	GGCCGCTTATATATATATAAAACAAAACAAGC	NotI

Supplementary Table S7: Primers for site-directed mutagenesis (SDM)

**Target Sites**	**Primer Sequences (5′-3′)**
MRE A	GAGGAGGAGAACTTCT**TGGTCG**AGCAGCAGCAGAGCGA
MRE C	AGGTGCCACGTCTCCACAC**TAGTCG**ACAACTACGCAGC
Rluc+ MRE A	TGCTGAAGAACGAGCAGCG**AGGAGGAGAACTTCTACCAGCA**TAATTCTAGGCG ATCGC
Rluc+ MYC CDS	TGCTGAAGAACGAGCAGCTCAACGTTAGCTTCAC

The bold font indicates the mutated nucleotides on the MRE sequence to which the miR-138 seed region binds. The SDM for MRE A and MRE B was performed on the ATG-less *MYC* CDS construct cloned into the psiCHECK-2 vector. The SDM for Rluc+ MRE A was performed to insert the MRE A (red bold font) into the *Renilla luciferase* gene, right before the stop codon. The SDM for Rluc+ MYC CDS was performed to fuse the Renilla luciferase gene and the ATG-less *MYC* CDS.
