# Supplementary material for: MiR-138 is a potent regulator of the heterogenous MYC transcript population in cancers
Source: Oncogene. 2021 Dec 22;41(8):1178–89. doi: 10.1038/s41388-021-02084-x (PMC8856960; doi:10.1038/s41388-021-02084-x)
Supplement: Supplementary file 3 — Supplementary Data File S1 [file 41388_2021_2084_MOESM3_ESM.pdf]

Supplementary Data File S1. MiRNAs detected in the *MYC* CDS MS2-TRAP

miRNA ID	Set A	
	MYC CDS	MYC 3'U
hsa-let-7a-2-3p	0.04	1.08
hsa-let-7a-3p	1.48	1.70
hsa-let-7a-5p	0.46	1.91
hsa-let-7b-3p	1.38	3.67
hsa-let-7b-5p	1.24	2.10
hsa-let-7d-3p	3.01	3.28
hsa-let-7d-5p	0.46	1.94
hsa-let-7e-3p	1.00	1.00
hsa-let-7e-5p	0.47	13.89
hsa-let-7f-1-3p	3.27	2.99
hsa-let-7f-2-3p	741.63	1114.21
hsa-let-7f-5p	0.12	1.14
hsa-let-7g-3p	942.93	11662.64
hsa-let-7g-5p	0.63	0.87
hsa-let-7i-5p	0.62	1.34
hsa-miR-100-5p	1.51	2.97
hsa-miR-101-3p	7.06	18.71
hsa-miR-101-5p	145.04	0.25
hsa-miR-103a-3p	1.22	2.12
hsa-miR-106a-3p	1.00	1.00
hsa-miR-106a-5p	1.99	3.41
hsa-miR-106b-3p	1.58	1.52
hsa-miR-106b-5p	2.87	2.90
hsa-miR-107	1.90	2.20
hsa-miR-10a-3p	1.01	4.84
hsa-miR-10a-5p	1.34	3.87
hsa-miR-1207-5p	0.48	1.93
hsa-miR-1224-3p	1765.62	1765.62
hsa-miR-1224-5p	1.21	4.39
hsa-miR-122-5p	1.00	1.00
hsa-miR-1226-3p	2.36	1.53
hsa-miR-125a-3p	0.56	2.67
hsa-miR-125a-5p	1.84	1.93
hsa-miR-125b-5p	1.42	2.08
hsa-miR-126-3p	1.31	3.07
hsa-miR-126-5p	1.91	7.19
hsa-miR-1271-5p	1.00	1.00
hsa-miR-1275	1.72	2.02
hsa-miR-1280 (v18)	2.45	1.82
hsa-miR-128-3p	2.11	2.45
hsa-miR-1285-3p	6.78	3.98
hsa-miR-1290	0.37	0.39
hsa-miR-1291	8.54	116.41
hsa-miR-1299	0.02	1.00
hsa-miR-130a-3p	3.62	4.50
hsa-miR-130b-3p	2.59	2.93
hsa-miR-130b-5p	0.95	2.29

miRNA ID	Set B	
	MYC CDS	MYC 3'U
hsa-let-7a-3p	1.15	0.38
hsa-let-7a-5p	0.19	0.22
hsa-let-7b-3p	0.99	0.60
hsa-let-7b-5p	0.77	1.51
hsa-let-7d-3p	1.64	1.05
hsa-let-7e-3p	0.51	2.11
hsa-let-7e-5p	0.15	0.76
hsa-let-7f-1-3p	1.14	0.98
hsa-let-7f-2-3p	186.80	2.31
hsa-let-7f-5p	1.29	0.98
hsa-let-7g-3p	0.21	0.01
hsa-let-7g-5p	0.56	0.71
hsa-let-7i-5p	0.45	0.65
hsa-miR-100-3p	10.51	2.28
hsa-miR-100-5p	0.63	0.23
hsa-miR-101-3p	0.11	0.22
hsa-miR-101-5p	0.00	0.66
hsa-miR-103a-2-5p	11.90	393862.97
hsa-miR-103a-3p	0.90	0.75
hsa-miR-106a-3p	6.14	394.81
hsa-miR-106a-5p	0.14	0.21
hsa-miR-106b-3p	0.17	0.44
hsa-miR-106b-5p	0.39	0.46
hsa-miR-107	0.68	0.76
hsa-miR-10a-3p	2.22	0.37
hsa-miR-10a-5p	1.26	0.38
hsa-miR-10b-3p	0.00	0.00
hsa-miR-10b-5p	1.88	0.38
hsa-miR-1204	0.58	25.79
hsa-miR-1224-3p	1.26	6.41
hsa-miR-1224-5p	0.21	0.35
hsa-miR-1238-3p	0.90	0.60
hsa-miR-1246	2.26	0.51
hsa-miR-1254	0.02	0.02
hsa-miR-1257	14.30	1070.15
hsa-miR-1258	4.09	0.66
hsa-miR-125a-3p	0.15	0.31
hsa-miR-125a-5p	0.70	0.25
hsa-miR-125b-1-3p	6.38	10.66
hsa-miR-125b-5p	1.74	0.84
hsa-miR-126-3p	2.28	0.48
hsa-miR-126-5p	1.14	0.51
hsa-miR-1271-5p	1.57	2.67
hsa-miR-1275	0.09	0.55
hsa-miR-1280 (v18)	0.31	0.12
hsa-miR-128-3p	0.78	0.61
hsa-miR-1285-3p	0.15	0.44

hsa-miR-132-3p	0.88	4.22
hsa-miR-133b	0.53	0.76
hsa-miR-134-5p	0.00	1.48
hsa-miR-135a-5p	0.12	1.53
hsa-miR-135b-5p	2.63	2.58
hsa-miR-139-5p	0.88	0.44
hsa-miR-1-3p	1.00	1.00
hsa-miR-140-3p	5.12	9.88
hsa-miR-141-3p	1.05	1.49
hsa-miR-142-3p	4.61	3.90
hsa-miR-142-5p	1.00	1.00
hsa-miR-143-3p	776.70	6.22
hsa-miR-145-5p	2.73	2.67
hsa-miR-146a-5p	6019.19	0.90
hsa-miR-146b-5p	2.59	1509.25
hsa-miR-148a-3p	2.32	2.20
hsa-miR-148a-5p	0.19	0.00
hsa-miR-148b-3p	2.70	2.82
hsa-miR-149-5p	1.77	1.83
hsa-miR-150-5p	1.00	0.00
hsa-miR-151a-3p	1.56	2.37
hsa-miR-151a-5p	1.96	5.89
hsa-miR-152-3p	1.68	0.20
hsa-miR-154-5p	1.00	1.00
hsa-miR-15a-3p	2.15	1.03
hsa-miR-15a-5p	2.34	2.32
hsa-miR-15b-3p	3.05	13.25
hsa-miR-15b-5p	1.35	2.42
hsa-miR-16-5p	1.12	1.71
hsa-miR-17-3p	4.58	3.13
hsa-miR-17-5p	1.52	3.46
hsa-miR-181a-2-3p	0.02	1.00
hsa-miR-181a-5p	0.91	2.23
hsa-miR-181b-5p	1.41	1.17
hsa-miR-181c-3p	40.58	36.32
hsa-miR-181c-5p	0.00	2.12
hsa-miR-181d-5p	0.48	0.95
hsa-miR-1825	1.00	1.00
hsa-miR-182-5p	0.75	1.40
hsa-miR-183-5p	0.61	1.96
hsa-miR-185-5p	2.38	1.50
hsa-miR-186-5p	3.54	4.83
hsa-miR-188-5p	1.63	2.16
hsa-miR-18a-3p	0.90	0.60
hsa-miR-18a-5p	0.00	0.00
hsa-miR-18b-5p	1.90	0.68
hsa-miR-191-5p	1.31	2.91
hsa-miR-192-5p	0.74	0.46
hsa-miR-193a-3p	0.28	0.39
hsa-miR-193a-5p	1.61	2.29

hsa-miR-1285-5p	0.97	1.57
hsa-miR-1290	0.63	1.29
hsa-miR-1291	0.51	0.04
hsa-miR-129-5p	0.67	6.45
hsa-miR-1296-5p	0.03	0.57
hsa-miR-1299	0.00	0.03
hsa-miR-1304-5p	4.42	5.16
hsa-miR-130a-3p	0.51	0.93
hsa-miR-130b-3p	0.39	0.44
hsa-miR-130b-5p	1.33	0.28
hsa-miR-132-3p	1.89	0.79
hsa-miR-133a-3p	2.10	2.35
hsa-miR-133b	32.98	0.08
hsa-miR-134-5p	159.75	51.32
hsa-miR-135a-5p	0.26	0.05
hsa-miR-135b-5p	1.07	0.58
hsa-miR-138-5p	0.13	0.45
hsa-miR-139-5p	4.51	1.06
hsa-miR-140-3p	0.16	0.53
hsa-miR-141-3p	0.27	0.37
hsa-miR-142-3p	3.65	1.35
hsa-miR-142-5p	0.51	0.25
hsa-miR-143-3p	0.65	0.72
hsa-miR-145-3p	236.03	0.00
hsa-miR-145-5p	1.63	0.81
hsa-miR-146a-5p	0.33	1.99
hsa-miR-146b-5p	1.52	0.49
hsa-miR-148a-3p	0.92	0.57
hsa-miR-148a-5p	0.72	1.05
hsa-miR-148b-3p	0.66	0.59
hsa-miR-148b-5p	0.20	0.22
hsa-miR-149-5p	0.67	2.78
hsa-miR-150-5p	3175.45	0.29
hsa-miR-151a-3p	0.32	0.67
hsa-miR-151a-5p	2.82	1.18
hsa-miR-152-3p	1.02	0.62
hsa-miR-153-3p	0.28	0.37
hsa-miR-154-3p	4.66	3.64
hsa-miR-15a-3p	0.06	3.83
hsa-miR-15a-5p	0.96	0.78
hsa-miR-15b-3p	1.11	0.79
hsa-miR-15b-5p	0.48	0.21
hsa-miR-16-5p	1.69	0.51
hsa-miR-17-3p	0.54	1.61
hsa-miR-17-5p	2.10	0.72
hsa-miR-181a-2-3p	0.36	0.00
hsa-miR-181a-5p	0.27	0.58
hsa-miR-181b-5p	0.17	0.38
hsa-miR-181c-3p	0.11	0.20
hsa-miR-181c-5p	0.35	0.62

hsa-miR-193b-3p	1.43	1.90
hsa-miR-194-5p	0.34	0.38
hsa-miR-195-5p	1.55	0.01
hsa-miR-196a-5p	0.49	1.86
hsa-miR-196b-5p	0.36	1.54
hsa-miR-1973	1.22	2.10
hsa-miR-197-3p	1.74	3.36
hsa-miR-199a-5p	0.68	0.93
hsa-miR-199b-3p	0.02	35.99
hsa-miR-199b-5p	0.06	3027.97
hsa-miR-19a-3p	1.33	3.11
hsa-miR-19b-3p	3.67	3.63
hsa-miR-200b-3p	0.77	1.79
hsa-miR-200b-5p	2.32	3.14
hsa-miR-200c-3p	1.69	3.21
hsa-miR-200c-5p	6.04	7.12
hsa-miR-203a-3p	1.07	1.08
hsa-miR-204-5p	1.00	1.00
hsa-miR-205-5p	2.56	0.38
hsa-miR-206	1.00	1.00
hsa-miR-20a-5p	2.24	4.01
hsa-miR-2113	1.04	0.61
hsa-miR-21-3p	2.17	1.87
hsa-miR-214-3p	1.57	931.72
hsa-miR-215-5p	1.00	188.11
hsa-miR-21-5p	2.61	4.32
hsa-miR-216a-5p	1065.88	37.56
hsa-miR-219a-5p	1.44	2.73
hsa-miR-221-3p	1.22	2.62
hsa-miR-221-5p	3.80	11.07
hsa-miR-222-3p	1.31	1.67
hsa-miR-223-3p	0.08	1.13
hsa-miR-22-3p	6.75	12.54
hsa-miR-224-5p	0.14	0.38
hsa-miR-22-5p	21.61	0.00
hsa-miR-2355-3p	0.71	100.59
hsa-miR-2355-5p	1.00	1.00
hsa-miR-23a-3p	1.96	1.30
hsa-miR-23b-3p	2.28	4.26
hsa-miR-23b-5p	2.13	0.00
hsa-miR-23c	1.00	1.00
hsa-miR-24-3p	2.02	3.00
hsa-miR-25-3p	1.13	2.08
hsa-miR-25-5p	0.70	0.85
hsa-miR-26a-2-3p	381.63	154.42
hsa-miR-26a-5p	1.00	1.00
hsa-miR-26b-5p	59570.36	59570.36
hsa-miR-27a-3p	1.75	0.96
hsa-miR-27a-5p	0.01	0.21
hsa-miR-27b-3p	1.95	2.31

hsa-miR-181d-5p	0.39	0.55
hsa-miR-182-3p	0.10	0.19
hsa-miR-182-5p	0.79	0.66
hsa-miR-183-5p	2.58	0.51
hsa-miR-184	0.16	0.10
hsa-miR-185-5p	0.60	0.59
hsa-miR-186-5p	0.27	0.90
hsa-miR-188-5p	1.15	3.09
hsa-miR-18a-3p	0.38	0.50
hsa-miR-18a-5p	0.29	0.69
hsa-miR-18b-5p	1.22	0.46
hsa-miR-1915-5p	0.17	0.34
hsa-miR-191-5p	0.46	0.52
hsa-miR-192-3p	5.94	4.36
hsa-miR-192-5p	0.52	0.67
hsa-miR-193a-3p	0.20	4.01
hsa-miR-193a-5p	1.54	0.76
hsa-miR-193b-3p	0.22	0.46
hsa-miR-193b-5p	0.02	0.21
hsa-miR-194-5p	0.18	0.53
hsa-miR-195-5p	0.97	0.24
hsa-miR-196a-5p	0.59	0.41
hsa-miR-196b-3p	0.02	0.05
hsa-miR-196b-5p	0.62	0.52
hsa-miR-1973	0.20	0.20
hsa-miR-197-3p	1.86	0.89
hsa-miR-199b-3p	3.04	0.38
hsa-miR-199b-5p	0.41	0.34
hsa-miR-19a-3p	0.56	0.41
hsa-miR-19b-1-5p	0.69	1.80
hsa-miR-19b-3p	0.49	0.46
hsa-miR-200b-3p	1.53	0.30
hsa-miR-200c-3p	1.55	0.31
hsa-miR-200c-5p	1.10	0.61
hsa-miR-203a-3p	0.43	0.30
hsa-miR-204-5p	4.49	0.28
hsa-miR-205-5p	0.51	0.49
hsa-miR-208b-3p	28.00	0.57
hsa-miR-20a-3p	2.59	0.27
hsa-miR-20a-5p	1.49	0.46
hsa-miR-212-3p	2.61	2.37
hsa-miR-21-3p	0.07	0.39
hsa-miR-214-3p	0.59	0.47
hsa-miR-215-5p	31676.92	3.26
hsa-miR-21-5p	0.97	0.29
hsa-miR-218-1-3p	5.52	0.31
hsa-miR-218-5p	1.00	54158.05
hsa-miR-219a-5p	0.00	0.10
hsa-miR-221-3p	0.14	0.41
hsa-miR-221-5p	1.71	0.70

hsa-miR-27b-5p	2.85	1.55
hsa-miR-28-3p	1.11	2.76
hsa-miR-28-5p	26.35	30.25
hsa-miR-297	##########	5067.65
hsa-miR-299-3p	58.27	1076.28
hsa-miR-29a-3p	1.46	2.64
hsa-miR-29a-5p	1.95	2.58
hsa-miR-29b-1-5p	1.75	2.61
hsa-miR-29b-2-5p	0.37	0.90
hsa-miR-29b-3p	3.99	3.87
hsa-miR-29c-3p	4.47	8.42
hsa-miR-29c-5p	100.94	169.35
hsa-miR-301a-3p	2.72	4.10
hsa-miR-301b-3p	103.75	2.78
hsa-miR-30a-3p	0.77	0.00
hsa-miR-30a-5p	1.00	318.63
hsa-miR-30b-3p	0.88	0.00
hsa-miR-30b-5p	3.18	2.62
hsa-miR-30c-5p	2.36	2.15
hsa-miR-30d-3p	64.50	64.50
hsa-miR-30d-5p	2.88	4.96
hsa-miR-30e-3p	0.51	2.61
hsa-miR-30e-5p	1.96	2.70
hsa-miR-320a	1.28	2.39
hsa-miR-320b	1.07	1.55
hsa-miR-320c	1.72	2.71
hsa-miR-320d	1.26	1.60
hsa-miR-320e	1.19	0.39
hsa-miR-323a-3p	0.88	0.61
hsa-miR-324-3p	1.69	4.46
hsa-miR-324-5p	1.82	1.43
hsa-miR-32-5p	1.44	4.43
hsa-miR-326	1.25	1.41
hsa-miR-330-3p	2.07	5.16
hsa-miR-331-5p	0.00	0.01
hsa-miR-335-3p	4.32	1.68
hsa-miR-335-5p	0.01	0.34
hsa-miR-337-5p	0.00	0.00
hsa-miR-338-3p	0.00	0.00
hsa-miR-338-5p	1.00	52.16
hsa-miR-339-3p	1.91	1.71
hsa-miR-339-5p	1.50	1.60
hsa-miR-33a-5p	1.85	4.04
hsa-miR-340-5p	14.09	21.76
hsa-miR-342-5p	0.52	11.39
hsa-miR-345-5p	1.24	1.84
hsa-miR-346	0.94	1.10
hsa-miR-34a-5p	1.75	2.08
hsa-miR-34b-5p	10.93	4.91
hsa-miR-361-5p	0.95	3.14

hsa-miR-222-3p	0.65	0.88
hsa-miR-223-3p	1.52	0.21
hsa-miR-22-3p	0.10	0.33
hsa-miR-224-5p	0.96	0.30
hsa-miR-22-5p	0.69	1.05
hsa-miR-2355-3p	15.11	1.12
hsa-miR-23a-3p	0.87	0.57
hsa-miR-23a-5p	0.16	0.64
hsa-miR-23b-3p	0.88	0.55
hsa-miR-23c	0.12	0.43
hsa-miR-24-3p	1.44	0.73
hsa-miR-25-3p	0.86	0.74
hsa-miR-25-5p	0.49	1.83
hsa-miR-26a-5p	1.27	0.51
hsa-miR-27a-3p	0.52	0.47
hsa-miR-27a-5p	0.41	0.23
hsa-miR-27b-3p	0.20	0.46
hsa-miR-27b-5p	4.53	0.38
hsa-miR-28-3p	0.50	0.57
hsa-miR-28-5p	1.97	0.57
hsa-miR-296-3p	59.46	19797.01
hsa-miR-296-5p	0.21	0.20
hsa-miR-297	14.19	3.51
hsa-miR-299-3p	0.03	9.34
hsa-miR-29a-3p	0.82	0.43
hsa-miR-29a-5p	0.43	0.74
hsa-miR-29b-2-5p	0.74	1.32
hsa-miR-29b-3p	0.05	0.18
hsa-miR-29c-3p	0.90	0.74
hsa-miR-29c-5p	0.47	0.73
hsa-miR-300	72.76	164.56
hsa-miR-301a-3p	1.04	0.85
hsa-miR-301b-3p	22.20	0.59
hsa-miR-302b-3p	0.70	1.08
hsa-miR-302c-5p	1.73	0.44
hsa-miR-302f	0.96	0.55
hsa-miR-30a-3p	15.76	17.32
hsa-miR-30b-5p	2.27	0.71
hsa-miR-30c-5p	1.62	0.50
hsa-miR-30d-3p	0.65	0.90
hsa-miR-30e-3p	0.45	0.36
hsa-miR-30e-5p	0.13	0.18
hsa-miR-320a	0.49	0.56
hsa-miR-320b	0.74	0.49
hsa-miR-320c	1.05	1.27
hsa-miR-320d	0.56	0.94
hsa-miR-320e	0.25	1.36
hsa-miR-323a-3p	3.93	12.88
hsa-miR-324-3p	0.30	2.28
hsa-miR-324-5p	0.50	0.75

hsa-miR-362-3p	5.22	1.88
hsa-miR-362-5p	4.18	6.54
hsa-miR-363-3p	1.00	1.00
hsa-miR-365a-3p	5.46	9.02
hsa-miR-365b-5p	0.01	0.00
hsa-miR-369-5p	1.00	1.00
hsa-miR-374a-3p	1.00	1.00
hsa-miR-374a-5p	1.05	6.32
hsa-miR-374b-5p	0.90	0.01
hsa-miR-374c-5p	420.52	4114.87
hsa-miR-375	1.91	2.40
hsa-miR-376b-3p	1.00	122.67
hsa-miR-377-3p	4.58	391.65
hsa-miR-378a-3p	1.19	1.84
hsa-miR-378a-5p	13.89	4.68
hsa-miR-378c	0.94	1.21
hsa-miR-379-3p	0.01	142.42
hsa-miR-379-5p	1.00	0.02
hsa-miR-381-3p	0.75	0.44
hsa-miR-382-5p	0.02	1.00
hsa-miR-411-3p	3422.23	36.33
hsa-miR-411-5p	0.76	0.76
hsa-miR-421	7.54	28.78
hsa-miR-423-5p	1.80	2.81
hsa-miR-424-5p	1.51	268.93
hsa-miR-425-3p	0.57	2.98
hsa-miR-425-5p	2.46	2.27
hsa-miR-4257	1.00	15.39
hsa-miR-4306	0.49	2517110.71
hsa-miR-431-5p	1.00	1.00
hsa-miR-449c-5p	1.77	3.55
hsa-miR-451a	0.10	0.19
hsa-miR-452-5p	1.90	0.30
hsa-miR-454-3p	3.23	8.28
hsa-miR-454-5p	1.00	0.53
hsa-miR-455-5p	1.30	1.73
hsa-miR-4732-3p	1.00	1.00
hsa-miR-483-3p	3.98	1.77
hsa-miR-483-5p	13.80	75.83
hsa-miR-484	2.12	1.88
hsa-miR-485-3p	60.71	60.71
hsa-miR-486-5p	0.44	0.40
hsa-miR-487a-3p	0.00	0.00
hsa-miR-487b-3p	1.00	1.00
hsa-miR-491-5p	0.60	1.90
hsa-miR-493-3p	0.01	0.01
hsa-miR-493-5p	0.89	1.00
hsa-miR-495-3p	6.32	6.32
hsa-miR-500a-3p	0.38	1.93
hsa-miR-500b-5p	0.00	2.51

hsa-miR-32-5p	0.24	0.97
hsa-miR-326	0.00	0.00
hsa-miR-328-3p	0.44	1.22
hsa-miR-330-3p	2.16	1.40
hsa-miR-330-5p	0.64	71.55
hsa-miR-331-5p	0.14	0.45
hsa-miR-335-3p	1.02	0.22
hsa-miR-335-5p	0.96	0.07
hsa-miR-338-3p	0.00	0.56
hsa-miR-339-3p	0.29	0.65
hsa-miR-33a-5p	0.01	0.39
hsa-miR-340-5p	2.06	0.31
hsa-miR-342-5p	2.20	2.72
hsa-miR-345-5p	0.58	0.94
hsa-miR-346	1.46	1.17
hsa-miR-34a-3p	0.32	0.39
hsa-miR-34a-5p	0.39	0.97
hsa-miR-34b-5p	12.95	0.51
hsa-miR-361-5p	1.26	0.36
hsa-miR-362-3p	1.16	0.15
hsa-miR-362-5p	1.30	0.50
hsa-miR-365a-3p	1.61	0.39
hsa-miR-365b-5p	0.93	0.60
hsa-miR-372-3p	2.45	60.24
hsa-miR-374a-3p	1.01	0.25
hsa-miR-374a-5p	1.73	0.28
hsa-miR-374b-5p	2.17	0.13
hsa-miR-374c-5p	3.55	0.89
hsa-miR-375	1.14	0.53
hsa-miR-376a-5p	6.27	43.89
hsa-miR-376b-3p	5.92	22.61
hsa-miR-377-3p	5010.33	0.46
hsa-miR-378a-3p	1.43	0.98
hsa-miR-378a-5p	0.12	0.32
hsa-miR-378c	0.85	1.94
hsa-miR-379-3p	4.90	103.41
hsa-miR-379-5p	0.12	0.61
hsa-miR-382-5p	0.63	0.32
hsa-miR-411-3p	0.01	0.14
hsa-miR-411-5p	1.02	4.25
hsa-miR-421	1.24	0.34
hsa-miR-423-5p	0.85	1.42
hsa-miR-424-5p	0.14	1.11
hsa-miR-425-3p	0.72	1.10
hsa-miR-425-5p	1.29	0.50
hsa-miR-429	1.71	0.46
hsa-miR-4306	112.87	0.67
hsa-miR-449c-3p	127.58	94.72
hsa-miR-449c-5p	0.45	0.42
hsa-miR-450a-5p	0.91	1.00

hsa-miR-501-3p	1.36	7.08
hsa-miR-501-5p	2.17	6.71
hsa-miR-502-3p	0.75	4.32
hsa-miR-503-5p	0.00	1.00
hsa-miR-505-3p	1.34	0.76
hsa-miR-532-3p	1.28	1.70
hsa-miR-532-5p	1.14	3.54
hsa-miR-539-5p	1.00	1.00
hsa-miR-542-5p	2.01	2.34
hsa-miR-550a-3p	1.13	1.32
hsa-miR-550a-5p	55.02	0.60
hsa-miR-551b-3p	0.01	0.01
hsa-miR-576-5p	191.42	2.25
hsa-miR-579-3p	1.00	1.00
hsa-miR-582-5p	3.78	0.54
hsa-miR-584-5p	1.27	1.45
hsa-miR-589-5p	1.39	1.52
hsa-miR-596	114.83	1.00
hsa-miR-597-5p	1.11	0.84
hsa-miR-598-3p	1.04	59.49
hsa-miR-616-3p	0.26	0.27
hsa-miR-616-5p	2.33	0.74
hsa-miR-620	1.00	2234.13
hsa-miR-625-5p	0.00	5.25
hsa-miR-627-5p	0.01	0.01
hsa-miR-628-5p	0.15	0.15
hsa-miR-629-3p	2.02	1.20
hsa-miR-629-5p	1.63	2.20
hsa-miR-651-5p	1.26	1703.37
hsa-miR-652-3p	0.87	1.18
hsa-miR-660-5p	6.95	6.42
hsa-miR-671-3p	0.74	3.85
hsa-miR-708-5p	1.23	0.85
hsa-miR-720 (v18)	4.99	1.66
hsa-miR-769-5p	0.43	1.82
hsa-miR-874-3p	1.00	1.00
hsa-miR-885-5p	0.15	0.36
hsa-miR-92a-3p	1.86	3.88
hsa-miR-92b-3p	1.39	2.44
hsa-miR-92b-5p	8.92	3.71
hsa-miR-93-3p	1.83	2.10
hsa-miR-9-3p	2.42	7.53
hsa-miR-95-3p	4.68	323.35
hsa-miR-96-5p	1.50	1.65
hsa-miR-99a-3p	0.21	0.39
hsa-miR-99a-5p	1.44	2.58
hsa-miR-99b-3p	1.62	1.77
hsa-miR-99b-5p	1.41	2.21

hsa-miR-451a	39.26	0.39
hsa-miR-452-5p	0.66	0.78
hsa-miR-454-3p	0.67	0.24
hsa-miR-454-5p	18.48	6.82
hsa-miR-455-5p	0.37	0.61
hsa-miR-4732-5p	1.52	3254.68
hsa-miR-483-3p	1.30	0.69
hsa-miR-483-5p	1.45	1.27
hsa-miR-484	12.12	1.35
hsa-miR-485-3p	0.44	0.45
hsa-miR-486-5p	0.80	1.55
hsa-miR-487a-3p	13.53	1.30
hsa-miR-487b-3p	1.10	0.32
hsa-miR-491-5p	0.00	0.27
hsa-miR-495-3p	0.01	0.07
hsa-miR-497-5p	0.47	0.69
hsa-miR-500a-3p	1.36	0.48
hsa-miR-500a-5p	0.41	0.55
hsa-miR-500b-5p	2.41	0.69
hsa-miR-501-3p	0.87	0.87
hsa-miR-501-5p	0.76	0.34
hsa-miR-502-3p	1.15	0.50
hsa-miR-503-5p	0.02	0.51
hsa-miR-505-3p	0.28	0.42
hsa-miR-508-5p	52.88	0.05
hsa-miR-515-3p	910.43	893.06
hsa-miR-517b-3p	0.06	3.98
hsa-miR-518f-5p	2.64	2.45
hsa-miR-525-5p	2.78	134.09
hsa-miR-532-3p	0.96	0.96
hsa-miR-532-5p	1.10	0.93
hsa-miR-539-5p	0.06	0.00
hsa-miR-542-5p	1.33	2.93
hsa-miR-550a-3p	3.18	6.12
hsa-miR-550a-5p	1.92	1.75
hsa-miR-551b-3p	0.09	0.24
hsa-miR-576-5p	0.73	0.20
hsa-miR-582-5p	6.26	1.75
hsa-miR-584-5p	0.65	0.45
hsa-miR-589-5p	0.40	1.23
hsa-miR-590-5p	0.48	0.62
hsa-miR-597-5p	0.66	0.78
hsa-miR-610	0.06	0.03
hsa-miR-616-3p	0.92	0.36
hsa-miR-616-5p	1.35	0.44
hsa-miR-618	0.01	0.08
hsa-miR-625-5p	1.29	1.05
hsa-miR-627-5p	0.10	0.47
hsa-miR-628-3p	3.02	2.85
hsa-miR-628-5p	5.61	0.65

hsa-miR-629-3p	1.40	0.81
hsa-miR-629-5p	0.58	1.33
hsa-miR-642b-3p	0.33	33.92
hsa-miR-651-5p	7.13	1.70
hsa-miR-652-3p	0.72	0.64
hsa-miR-660-5p	1.59	1.04
hsa-miR-664a-3p	0.89	0.21
hsa-miR-668-3p	31.65	605.97
hsa-miR-671-3p	0.92	1.45
hsa-miR-708-5p	4.71	10.63
hsa-miR-7-1-3p	2.12	0.71
hsa-miR-720 (v18)	1.08	0.10
hsa-miR-769-5p	0.14	0.56
hsa-miR-92a-3p	4.65	1.34
hsa-miR-92b-3p	1.10	0.64
hsa-miR-92b-5p	0.01	0.18
hsa-miR-93-3p	0.63	0.83
hsa-miR-9-3p	0.89	0.14
hsa-miR-95-3p	2.80	1.27
hsa-miR-9-5p	0.00	0.64
hsa-miR-96-5p	0.20	1.01
hsa-miR-99a-3p	23.24	107.72
hsa-miR-99a-5p	0.89	0.27
hsa-miR-99b-3p	1.51	1.01
hsa-miR-99b-5p	1.49	0.67

Supplementary Data File S1. MiRNAs detected in the MYC CDS MS2-TRAP.

Four miRNAs, miR-184, miR-744, miR-320a, and miR-320b, have been validated to regulate the MYC CDS. MiR-184 and miR-320a were only detected in one set of the pulldowns, but not enriched in the MYC CDS pulldown. MiR-320b was detected in both sets, but not enriched in the MYC CDS. MiR-744 was not in the list of 500 detected miRNAs. Five miRNAs were shortlisted from the miRNA target prediction, miR-125a-3p, miR-138, miR-139-3p, miR-503-5p and miR-744. miR-125a-3p and miR-503-5p were enriched in one set of the pulldowns, while miR-138 and miR-139-3p were not detected in both sets.
