# Supplementary material for: MiR-138 is a potent regulator of the heterogenous MYC transcript population in cancers
Source: Oncogene. 2021 Dec 22;41(8):1178–89. doi: 10.1038/s41388-021-02084-x (PMC8856960; doi:10.1038/s41388-021-02084-x)
Supplement: Supplementary file 4 — Supplementary Data File S2 [file 41388_2021_2084_MOESM4_ESM.pdf]

MicroRNA and Target Gene Description:

**miRNA Name**	hsa-miR-138-5p	**miRNA Sequence**	AGCUGGUGUUGUGAAUCAGGCCG
**Previous Name**	hsa-miR-138		
**Target Score**	57	**Seed Location**	95, 741, 1026
**Target Length**	1320		

Custom Target Sequence

1 atgcccctca acgttagctt caccaacagg aactatgacc tcgactacga ctcggtgcag
61 ccgtatttct actgcgacga ggaggagaac ttct**accagc a**gcagcagca gagcgagctg
121 cagcccccgg cgcccagcga ggatatctgg aagaaattcg agctgctgcc caccccgccc
181 ctgtccccta gccgccgctc cgggctctgc tcgccctcct acgttgcggt cacacccttc
241 tcccttcggg gagacaacga cggcggtggc gggagcttct ccacggccga ccagctggag
301 atggtgaccg agctgctggg aggagacatg gtgaaccaga gtttcatctg cgacccggac
361 gacgagacct tcatcaaaaa catcatcatc caggactgta tgtggagcgg cttctcggcc
421 gccgccaagc tcgtctcaga gaagctggcc tcctaccagg ctgcgcgcaa agacagcggc
481 agcccgaacc ccgcccgcgg ccacagcgtc tgctccacct ccagcttgta cctgcaggat
541 ctgagcgccg ccgcctcaga gtgcatcgac ccctcggtgg tcttccccta ccctctcaac
601 gacagcagct cgcccaagtc ctgcgcctcg caagactcca gcgccttctc tccgtcctcg
661 gattctctgc tctcctcgac ggagtcctcc ccgcagggca gccccgagcc cctggtgctc
721 catgaggaga caccgcccac **caccagca**gc gactctgagg aggaacaaga agatgaggaa
781 gaaatcgatg ttgtttctgt ggaaaagagg caggctcctg gcaaaaggtc agagtctgga
841 tcaccttctg ctggaggcca cagcaaacct cctcacagcc cactggtcct caagaggtgc
901 cacgtctcca cacatcagca caactacgca gcgcctccct ccactcggaa ggactatcct
961 gctgccaaga gggtcaagtt ggacagtgtc agagtcctga gacagatcag caacaaccga
1021 aaatg**cacca gc**cccaggtc ctcggacacc gaggagaatg tcaagaggcg aacacacaac
1081 gtcttggagc gccagaggag gaacgagcta aaacggagct tttttgccct gcgtgaccag
1141 atcccggagt tggaaaacaa tgaaaaggcc cccaaggtag ttatccttaa aaaagccaca
1201 gcatacatcc tgtccgtcca agcagaggag caaaagctca tttctgaaga ggacttgttg
1261 cggaaacgac gagaacagtt gaaacacaaa cttgaacagc tacggaactc ttgtgcgtaa

accagca – Site 1

caccagca – Site 2

caccagc – Site 3

Supplementary Data File S2. Predicted miR-138 binding on the human *MYC* CDS by miRDB

Three MREs were predicted and indicated in blue font. The first MRE is the MRE A which is also predicted by RNA22 and STarMir.
